# Mechanisms and efficacy of small molecule latency-promoting agents to inhibit HIV reactivation ex vivo

**DOI:** 10.1172/jci.insight.183084

**Published:** 2024-08-20

**Authors:** Julie Janssens, Peggy Kim, Sun Jin Kim, Adam Wedrychowski, Gayatri N. Kadiyala, Peter W. Hunt, Steven G. Deeks, Joseph K. Wong, Steven A. Yukl

**Affiliations:** 1 Department of Medicine, University of California San Francisco (UCSF), San Francisco, California, USA.; 2 Department of Medicine, San Francisco Veterans Affairs Medical Center, San Francisco, California, USA.

**Keywords:** Virology, Drug screens, Transcription

## Abstract

Drugs that inhibit HIV transcription and/or reactivation of latent HIV have been proposed as a strategy to reduce HIV-associated immune activation or to achieve a functional cure, yet comparative studies are lacking. We evaluated 26 drugs, including drugs previously reported to inhibit HIV transcription (inhibitors of Tat-dependent HIV transcription, Rev, HSF-1/PTEF-b, HSP90, Jak/Stat, or SIRT1/Tat deacetylation) and other agents that were not tested before (inhibitors of PKC, NF-κB, SP-1, or histone acetyltransferase; NR2F1 agonists), elongation (inhibitors of CDK9/ PTEF-b), completion (inhibitors of PolyA-polymerase), or splicing (inhibitors of human splice factors). To investigate if those drugs would vary in their ability to affect different blocks to HIV transcription, we measured levels of initiated, elongated, midtranscribed, completed, and multiply spliced HIV RNA in PBMCs from antiretroviral therapy–suppressed individuals following ex vivo treatment with each drug and subsequent T cell activation. We identified new drugs that prevent HIV reactivation, including CDK and splicing inhibitors. While some drugs inhibited 1 or 2 steps, other drugs (CDK inhibitors, splicing inhibitors, tanespimycin, and triptolide) inhibited multiple stages of HIV transcription and blocked the production of supernatant viral RNA. These drugs and targets deserve further study in strategies aimed at reducing HIV-associated immune activation or achieving a functional cure.

## Abstract 2

HIV infection cannot be cured due to the persistence of a reservoir of latently infected cells. Furthermore, virally suppressed individuals experience chronic immune activation from ongoing low-level viral expression. Drugs that inhibit HIV transcription and/or reactivation of latent HIV have been proposed as a strategy to reduce HIV-associated immune activation and/or to achieve a functional cure. We evaluated 26 small molecules, both previously reported drugs and new drug candidates, for their ability to act as “latency promoting/silencing agents (LPAs)” that can reduce or prevent HIV expression after T cell activation. Using a panel of RT-ddPCR assays, we measured the progression through HIV transcription and pinpointed the step at which each of those drugs inhibited HIV transcription, with and without prior activation. While some drugs primarily inhibited one or two steps in HIV reactivation, other drugs (CDK inhibitors, splicing inhibitors, tanespimycin, and triptolide) inhibited multiple stages of HIV transcription and blocked the production of supernatant viral RNA. Dinaciclib, AZD4573, and pladienolide B also appeared to inhibit HIV splicing in unstimulated PBMC. By selecting drugs with known mechanisms of action, we specifically identified cellular factors and pathways that may be involved in regulation of HIV expression. These drugs/targets deserve further study in strategies aimed at reducing HIV-associated immune activation or achieving a functional cure.

## Introduction

The global number of HIV-related deaths has been strikingly reduced by antiretroviral therapy (ART). Nonetheless, ART fails to cure HIV, since HIV persists as a reservoir of infectious proviruses, including latently infected CD4^+^ T cells that do not produce virions constitutively but can be induced to produce infectious virus after physiologic T cell activation (1–3). The reversible silencing of viral gene expression allows survival of latently infected cells, while reactivation from a small subset of these cells is thought to be responsible for the plasma virus that rebounds after ART interruption (4, 5). Preventing HIV rebound from intact, replication-competent reservoirs will be an absolute prerequisite and poses the main barrier to cure HIV infection.

Cell-associated HIV transcripts can be detected in almost all people with HIV (PWH) on ART, despite suppression of plasma virus (6–9). Antiretroviral drugs in current clinical use do not block HIV transcription, providing a mechanism for the persistence of HIV-transcribing cells on prolonged ART. Using a panel of assays that can simultaneously quantify multiple different regions of HIV RNA, we recently showed that most infected CD4^+^ T cells from the blood of ART-suppressed PWH have initiated HIV transcription, while successively smaller fractions express 5′ elongated, polyadenylated (completed), and multiply spliced HIV transcripts, likely because of reversible blocks at sequential stages of HIV transcription/splicing (8, 10). While many of the proviruses in this “active reservoir” may be defective (9), some of the defective HIV RNA may trigger intracellular pattern recognition receptors and/or be translated into viral proteins that contribute to immune activation. The mechanisms of chronic immune activation in PWH are not completely defined and may be multifactorial, but the persistence of cell-associated HIV RNA (reviewed in ref. 11) has been correlated with T cell activation (12). It is believed that residual immune activation contributes to “non-AIDS” morbidity and mortality observed in ART-treated PWH (13).

These findings advocate for the development of therapies that can block reactivation from latency or inhibit HIV transcription in order to curb immune activation in ART-treated PWH. Additionally, inhibitors that durably silence HIV transcription could be beneficial in a “block-and-lock” strategy aimed at functional HIV cure (14). The objective of this strategy is to reinforce latency and prevent HIV reactivation in order to achieve a state of HIV remission without the need for ART. An example of a promising block-and-lock strategy is the inhibition of the HIV transcription factor Tat using didehydro-cortistatin A (15) since Tat is needed to recruit PTEF-b and ensure efficient elongation of HIV transcription. In addition to Tat, HIV relies heavily on cellular factors to ensure robust transcription from the long terminal repeat (LTR) promoter.

As such, cellular pathways imposing restrictions at the HIV promoter and elongation phase present interesting targets to silence HIV expression. For example, the mTOR inhibitor rapamycin reduced basal transcription (16) but not activation-induced HIV transcription (17), while INK128 reduced both via inhibition of PKC and downstream NF-κB signaling (18). In addition, inhibition of HSP90 has been reported to durably block HIV reactivation in vitro (19) and in vivo (20), even after the removal of the drug, by impacting NF-κB, NFAT, and STAT5 signaling pathways. Alternatively, the Jak-Stat inhibitor ruxolitinib reduced viral reactivation by blocking cytokine-induced STAT signaling (21). Recently, filgotinib was shown to reduce HIV splicing through a mechanism known as intron retention (22), a process previously observed for topotecan, another latency-promoting agent (23, 24). Alternatively, HIV can be silenced epigenetically (25–27), including RNA-induced epigenetic silencing (28–30), or by retargeting the provirus out of active chromatin into transcriptionally silent regions (31, 32).

In this study, we examined both previously reported drugs and new drug candidates for their ability to act as latency-promoting/-silencing agents (LPAs) that can reduce or prevent HIV expression after T cell activation. We screened 26 molecules from various categories, including 1) agents previously reported to inhibit HIV transcription (such as inhibitors of Tat-dependent HIV transcription, Rev, HSF-1/PTEF-b, and HSP90) and/or T cell activation (Jak-Stat inhibitors); 2) drugs with mechanisms opposite of previously reported latency-reversing agents (PKC inhibitors); and 3) drugs predicted to inhibit HIV transcription initiation (inhibitors of NF-κB, SP-1, or histone acetyltransferase; NR2F1 agonists), elongation (inhibitors of CDK9/PTEF-b or SIRT1/Tat deacetylation), completion (inhibitors of PolyA-polymerase), or splicing (inhibitors of human splice factors) (overview in Supplemental Table 1; supplemental material available online with this article; https://doi.org/10.1172/jci.insight.183084DS1). Many of the selected drugs are either in clinical use or in human trials.

The aims of this study were to use drugs as mechanistic probes to inform us about cellular pathways involved in HIV latency/reactivation, to identify new LPAs, and to compare the efficacy of various LPAs in cells from PWH. We hypothesized that drugs acting through different mechanisms would act selectively to impair the activation-induced reversibility of blocks at various stages of HIV transcription (or enhance baseline blocks in unstimulated cells). To investigate this hypothesis, we measured levels of different HIV RNA regions/transcripts in PBMCs from ART-suppressed individuals following ex vivo treatment with different drugs (or control) with or without subsequent T cell activation. Various drugs selectively blocked activation-induced increases in HIV transcriptional initiation, elongation, midtranscription, completion, or splicing. We discovered new LPAs, such as CDK inhibitors and splicing inhibitors, with nanomolar potency. Some drugs (including the CDK9 inhibitor AZD4573, the splicing inhibitor pladienolide B, the HSP90 inhibitor tanespimycin, and triptolide) inhibited multiple stages of HIV transcription and blocked the production of supernatant viral RNA (schematic overview in Supplemental Figure 1). These drugs and combinations should be investigated further as LPAs aimed to curb immune activation or toward a functional cure.

## Results

### Latency reversal assay in cells from PWH reveals candidate LPAs.

**Figure 1:**
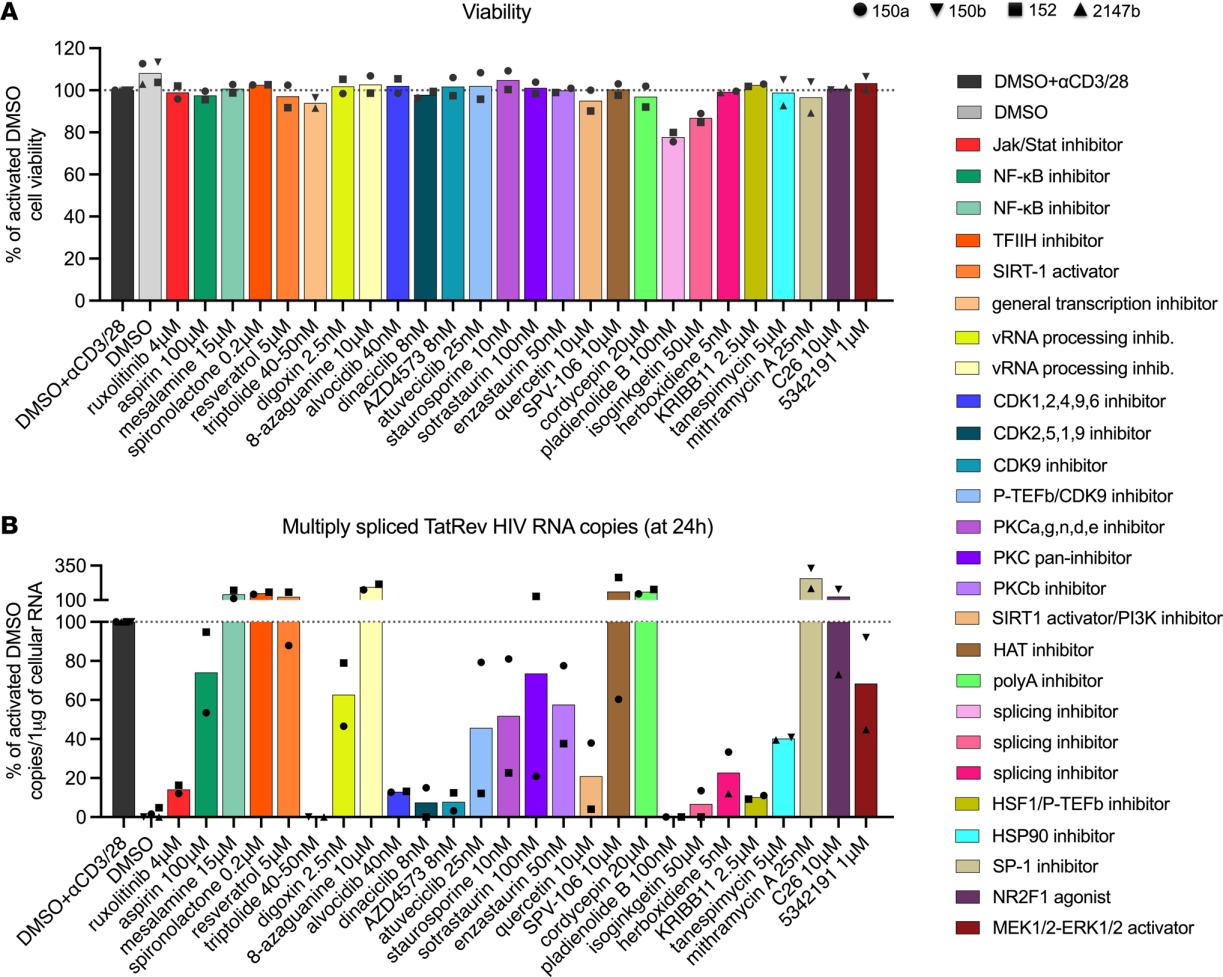
Screening of candidate LPAs by measuring the change in multiply spliced HIV transcripts after activation. Each drug was tested in PBMCs from 2 ART-suppressed study participants (denoted in the legend by varying symbol shapes). PBMCs were aliquoted into wells at 6 × 10^6^ cells/well. After activation, the cells were cultured with antiretrovirals in the presence of individual drugs in DMSO or DMSO alone as control. All conditions were tested in the presence of CD3/28 T cell–activating beads, except for the unactivated DMSO condition. (**A**) After 24 hours, the viability was measured by trypan blue staining and then normalized to the levels of the activated DMSO (% of activated DMSO). Bars indicate medians. (**B**) Total cellular RNA was extracted, and the levels of multiply spliced HIV transcripts (TatRev) were measured by RT-ddPCR, normalized to 1 μg of total cellular RNA, and expressed as a percentage of the activated DMSO control (% of activated DMSO). RT-dd, reverse transcription digital droplet; vRNA, viral RNA; HAT, histone acetyltransferase.

We selected 26 small molecules targeting pathways implicated in HIV transcription or the blocks to HIV transcription underlying latency (Supplemental Table 1). As positive controls, we included drugs such as ruxolitinib (21, 33) and KRIBB11 (34) for their well-characterized inhibition of HIV expression (didehydro-cortistatin A [ref. 15] could not be obtained). Initial drug concentrations were chosen based on levels attainable in plasma (when known) or set at twice the IC_50_ for the target of the drug and subsequently adjusted to the lowest effective and nontoxic dose. Initial screening of all drugs was performed in freshly isolated PBMCs from 2 ART-suppressed individuals (Figure 1 and Supplemental Figure 2). PBMCs were cultured in the presence of individual drugs in DMSO or DMSO alone as a control, subsequently activated (anti-CD3/CD28), and harvested after 24 hours. Most drugs had no impact on cell viability (Figure 1A), except triptolide, pladienolide B, and isoginkgetin. As a result, the doses of those 3 drugs were subsequently reduced. The progression through different blocks to HIV transcription was quantified by measuring the levels of initiated (TAR), 5′ elongated (LLTR), midtranscribed/unspliced (Pol), completed (PolyA), and multiply spliced (TatRev) HIV transcripts. The levels of those HIV transcripts were normalized to 1 μg of total cellular RNA (to normalize for the effect of the drug on global cellular transcription) and expressed as a percentage of the activated DMSO control.

Triptolide profoundly reduced elongated (median relative to activated DMSO: 21%), midtranscribed (9%), and multiply spliced (0%) HIV transcripts (Supplemental Figure 2A). CDK inhibitors reduced elongated HIV transcripts (median relative to activated DMSO between 67% and 76%) and further reduced midtranscribed (median 20% and 43%) and multiply spliced HIV transcripts (8% and 13%), except for atuveciclib (46%, Supplemental Figure 2C). Splicing inhibitors induced a modest reduction in midtranscribed HIV RNA (52% and 77%) but further reduced completed (2% for isoginkgetin, 17% for pladienolide B) and multiply spliced HIV transcripts (23% for herboxidiene, Supplemental Figure 2D). PKC inhibitors were most effective in reducing midtranscribed HIV transcripts (51%–68%), though staurosporine and sotrastaurin also reduced completed HIV transcripts (both 41%, Supplemental Figure 2E).

Of the signal transduction inhibitors, ruxolitinib (Jak-Stat inhibitor), KRIBB11 (HSF-1/PTEF-b inhibitor), and tanespimycin (HSP90 inhibitor) reduced elongated HIV transcripts (medians relative to activated DMSO: 39%, 60%, and 49%, respectively) and midtranscribed HIV transcripts (14%, 16%, and 31%, respectively), but no additional decrease was observed in completed or multiply spliced HIV transcripts (Supplemental Figure 2H). In contrast, quercetin (SIRT1 activator/PI3K inhibitor) had no effect on elongated transcripts but induced progressive reductions in midtranscribed (57%), completed (41%), and multiply spliced HIV transcripts (21%, Supplemental Figure 2H). No substantial reductions in HIV transcripts were observed for inhibitors of NF-κB, viral RNA processing, SP-1, or histone acetyltransferase (Supplemental Figure 2, B, F, and I). Since multiply spliced HIV transcripts are used as a predictive marker for productive infection after latency reversal (35, 36), drugs that reduced multiply spliced or completed HIV transcripts by more than 50% were selected from our drug screen as candidate LPAs for further experiments (i.e., ruxolitinib, triptolide, alvocidib, dinaciclib, AZD4573, quercetin, pladienolide B, isoginkgetin, herboxidiene, KRIBB11, and tanespimycin; Figure 1B).

### Dose-response curves demonstrate LPAs with nanomolar potency.

**Figure 2:**
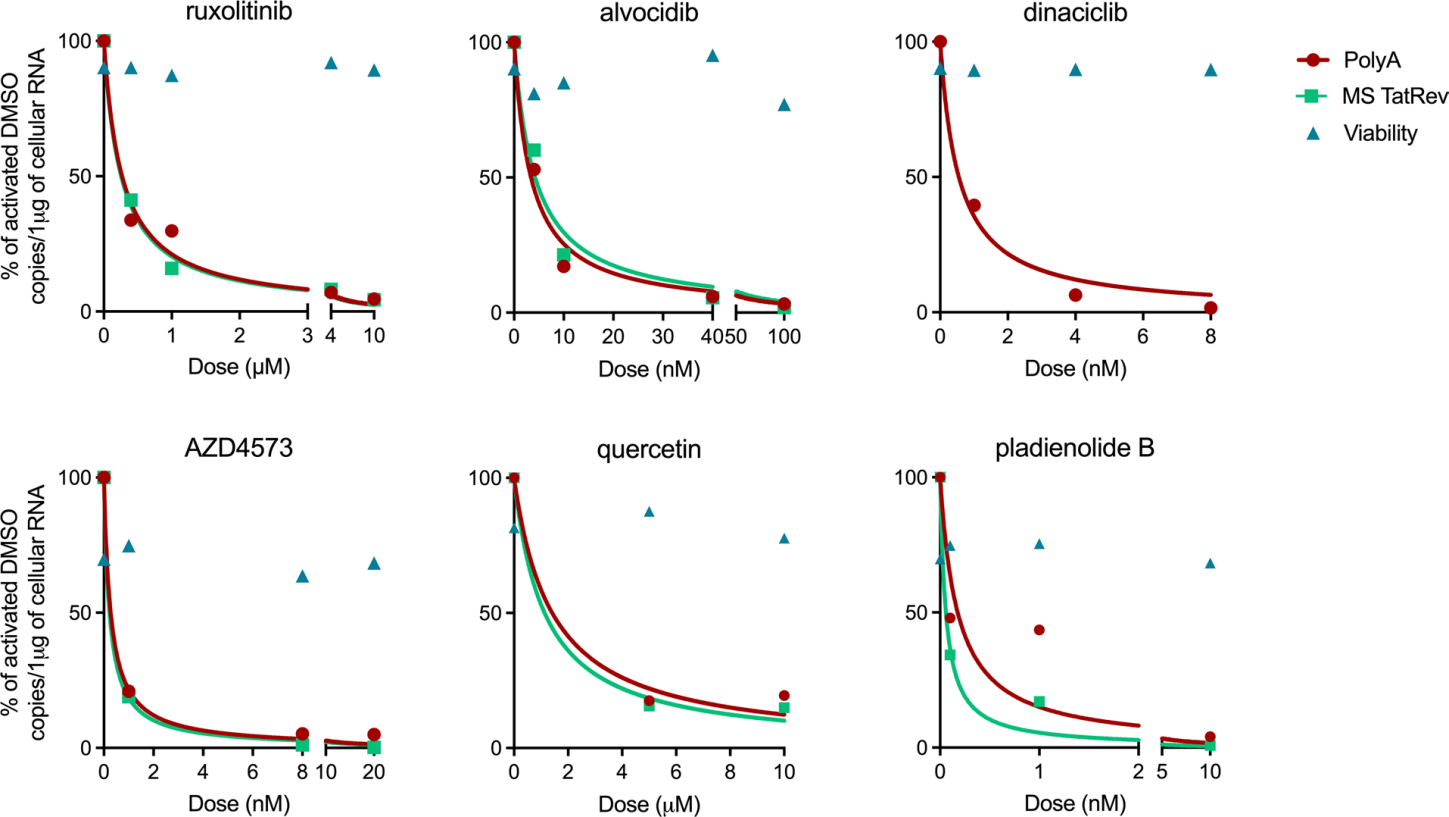
Dose-response curves of LPAs. Dose-response curves for each drug were generated in PBMCs from 1 ART-suppressed study participant per drug. PBMCs were aliquoted into wells at 6 × 10^6^ cells/well, activated, and cultured with antiretrovirals in the presence of DMSO alone (no drug) or varying concentrations of individual drugs in DMSO. After 24 hours, the levels of polyadenylated and multiply spliced HIV transcripts were quantified by RT-ddPCR, normalized to 1 μg of total cellular RNA, and expressed as a percentage of the activated DMSO control (% of activated DMSO). The IC_50_ was determined for ruxolitinib, alvocidib, dinaciclib, AZD4573, quercetin, and pladienolide B using nonlinear regression in Prism.

We examined dose-response curves for ruxolitinib, CDK inhibitors (alvocidib, dinaciclib, and AZD4573), quercetin, and pladienolide B (Figure 2). The IC_50_ was determined based on the reduction in completed (PolyA) as well as multiply spliced (TatRev) HIV transcripts relative to activated DMSO (Table 1). We obtained low micromolar IC_50_ for ruxolitinib (0.27 and 0.26 μM for PolyA and TatRev transcripts, respectively). For CDK inhibitors, we obtained nanomolar IC_50_ for alvocidib (3.41 and 4.23 nM, respectively) and low nanomolar IC_50_ for AZD4573 (0.28 and 0.23 nM) and dinaciclib (0.55 nM). The most potent IC_50_ was observed for pladienolide B (0.18 and 0.06 nM for PolyA and TatRev transcripts, respectively). Quercetin demonstrated an IC_50_ of 1.41 μM and 1.13 μM for PolyA and TatRev transcripts, respectively. Due to possible decreases in viability at 10 μM, its concentration was reduced to 5 μM for subsequent experiments.

### Various LPAs affect distinct blocks to HIV transcription after activation.

**Figure 3:**
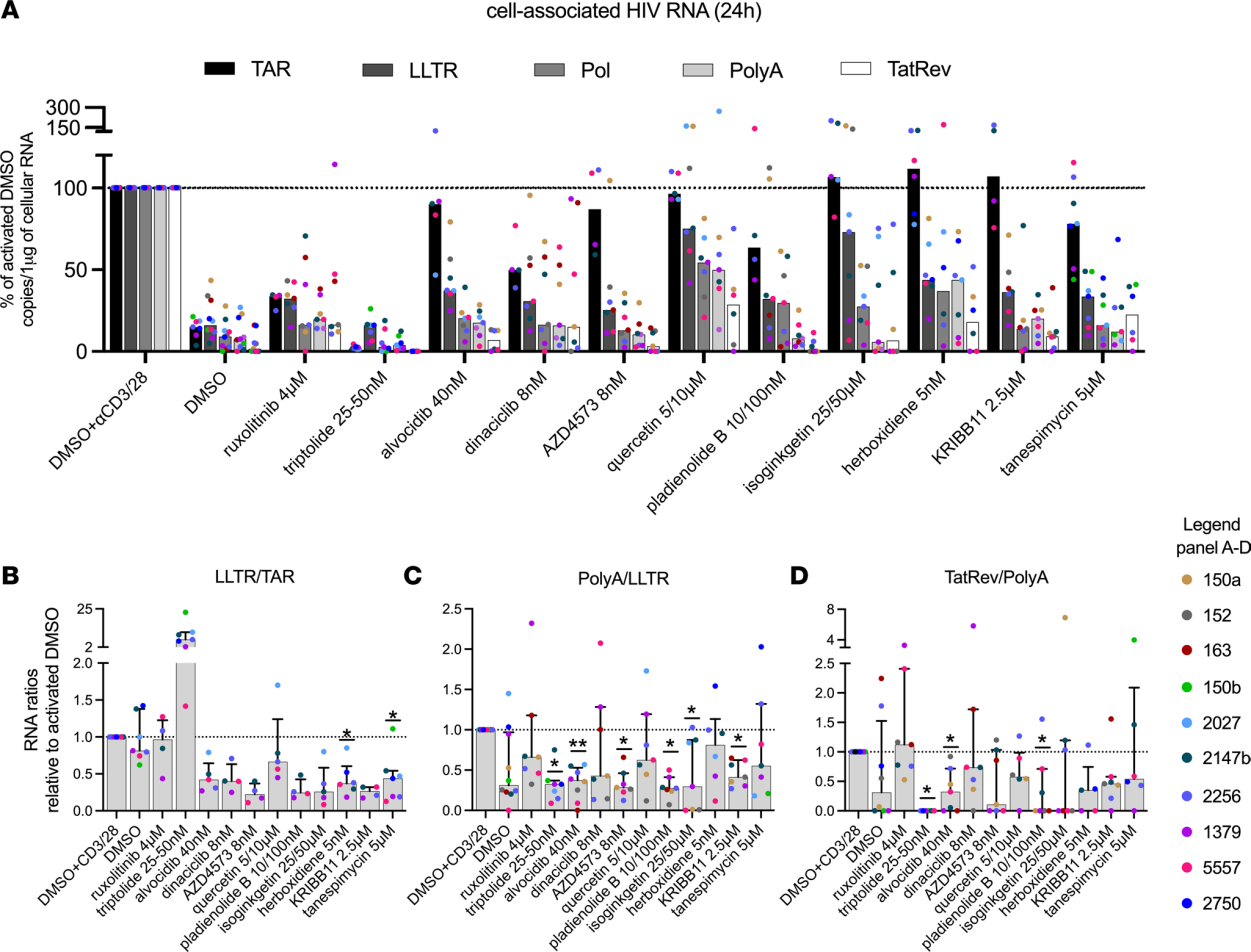
LPAs block HIV reactivation in freshly isolated PBMCs from PWH by reducing transcriptional initiation, elongation, completion, or splicing after activation. Each drug was tested in PBMCs from 7 ART-suppressed study participants. The PBMCs were aliquoted into wells at 6 × 10^6^ cells/well. After activation, the cells were cultured with antiretrovirals in the presence of individual drugs in DMSO or DMSO alone as control. All conditions were tested in the presence of CD3/28 T cell–activating beads, except for the unactivated DMSO condition. After 24 hours, total RNA was extracted, and the progression through different stages of HIV transcription was quantified by measuring the levels of initiated (TAR), 5′ elongated (LLTR), midtranscribed/unspliced (Pol), completed (PolyA), and multiply spliced (TatRev) HIV transcripts. (**A**) The levels of all HIV transcripts were normalized to 1 μg of total cellular RNA and expressed as a percentage of the activated DMSO control (% of activated DMSO). Medians are shown as well as the individual values per study participant in different colors. (**B**–**D**) The effect of each drug on HIV transcriptional progression after activation was analyzed by the ratio of one HIV transcript to another. Ratios are independent of HIV infection frequency or normalization to cell numbers. Shown are the proportion of (**B**) all HIV transcripts that were elongated (LLTR/TAR), (**C**) elongated HIV transcripts that were completed spliced (PolyA/LLTR), and (**D**) completed transcripts that were multiply spliced (TatRev/PolyA). Medians and IQR are presented, as well as the individual values per study participant in different colors. *P* values were calculated using Wilcoxon’s signed-rank test: *P < 0.05; **P < 0.01.

The ability of our candidate LPAs to inhibit HIV transcriptional progression after activation was examined in PBMCs from a minimum of 7 PWH per drug tested (Figure 3). PBMCs were cultured in the presence of individual drugs in DMSO or in DMSO alone as a control, subsequently activated (anti-CD3/CD28), and harvested after 24 hours. Only ruxolitinib and triptolide reduced initiated HIV transcripts (Figure 3A and Table 2; median relative to activated DMSO: 34% and 2%, respectively; *P* = 0.02 for triptolide). Ruxolitinib further reduced midtranscribed HIV RNA (16%, *P* = 0.02), but no additional reductions were observed in completed or multiply spliced HIV transcripts, in line with previous data (33). The CDK inhibitors alvocidib, dinaciclib, and AZD4573 potently reduced elongated (37%, 31%, and 25%, respectively; all *P* = 0.02, except *P* = 0.03 for AZD4573), midtranscribed (20%, 16%, and 13%; all *P* = 0.02), and multiply spliced HIV RNA (7%, 15%, and 3%; all *P* = 0.02, except *P* = 0.03 for alvocidib). Quercetin had little effect on initiated or elongated HIV RNA but significantly reduced midtranscribed (54%; *P* = 0.02) and multiply spliced HIV RNA (29%; *P* = 0.03).

Of the splicing inhibitors, pladienolide B and herboxidiene reduced elongated HIV RNA (Figure 3A and Table 2; median relative to activated DMSO: 32% and 44%, respectively; *P* = 0.02 for herboxidiene), while isoginkgetin had less effect on elongated HIV RNA but instead reduced midtranscribed HIV RNA (28%; *P* = 0.02). Pladienolide B and isoginkgetin both potently reduced completed HIV RNA (8% and 6%, respectively; both *P* = 0.02), while pladienolide B and herboxidiene further reduced multiply spliced HIV transcripts (0%, *P* = 0.02 and 18%, *P* = 0.03, respectively). KRIBB11 and tanespimycin both reduced elongated HIV RNA (36% and 34%, respectively; both *P* = 0.02) and midtranscribed HIV RNA (14% and 16%; both *P* = 0.02), but only KRIBB11 was able to further reduce multiply spliced HIV transcripts (9%; *P* = 0.02). Most of the effects on 5′ elongation, midtranscription, completion, and splicing remained significant even after correcting for multiple comparisons (Benjamini-Hochberg method; Table 2 and Supplemental Data 1). When analyzing the effects of the drugs in individual study participants, we observed consistent reductions in 5′ elongated, midtranscribed, completed, and multiply spliced HIV transcripts, with only a few exceptions where specific drugs did not show an effect in a certain participant (Supplemental Figure 3).

We also calculated the ratio of one HIV RNA to another, allowing us to evaluate the progression through HIV transcriptional elongation, completion, and splicing independent of effects at prior stages of HIV transcription and independent of infection frequency or normalization to cell numbers. HIV transcriptional elongation (ratio of 5′ elongated to initiated HIV transcripts [LLTR/TAR]; Figure 3B) was inhibited by CDK inhibitors, splicing inhibitors, KRIBB11, and tanespimycin as compared with the activated DMSO. Significant reductions were obtained for herboxidiene and tanespimycin (both *P* = 0.03), and trends were observed for alvocidib and isoginkgetin (both *P* = 0.06). Interestingly, although ruxolitinib and triptolide dramatically reduced HIV transcriptional initiation (Figure 3A, Supplemental Figure 3A, and Table 2), they had no impact on the subsequent elongation phase (Figure 3B).

HIV transcriptional completion (ratio of polyadenylated to 5′ elongated HIV RNA [PolyA/LLTR]; Figure 3C) was reduced for the CDK inhibitors alvocidib and AZD4573 (*P* = 0.008 and *P* = 0.02, respectively) but not for dinaciclib. Completion was also reduced for the splicing inhibitors pladienolide B (*P* = 0.02) and isoginkgetin (*P* = 0.03) but not for herboxidiene. KRIBB11 and triptolide also reduced completion (both *P* = 0.02). Finally, only a few drugs were able to reduce HIV multiple splicing (ratio of multiply spliced to completed HIV RNA [TatRev/PolyA]; Figure 3D), including alvocidib (*P* = 0.02), triptolide (*P* = 0.03), and pladienolide B (*P* = 0.046). After correcting for multiple testing, only the effects on completed transcripts remained significant for all the drugs, except for isoginkgetin (Figure 3C and Supplemental Data 1).

### Some LPAs sustain reduced HIV transcription for 6 days, depending on the study participant.

**Figure 4:**
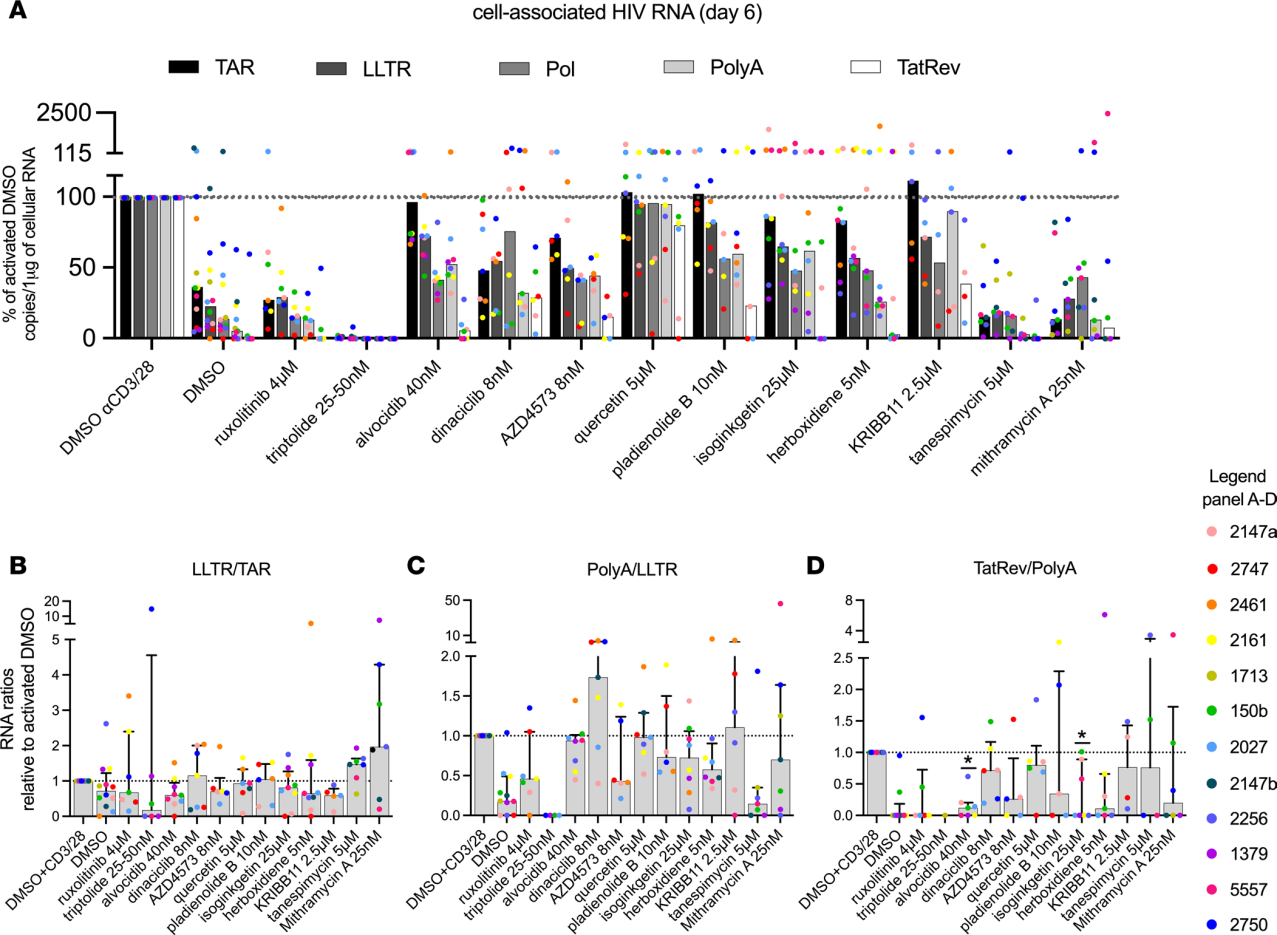
Some LPAs sustain reduced HIV transcription for 6 days, depending on the study participant. Each drug was tested in PBMCs from at least 6 ART-suppressed study participants (except for KRIBB11, *n* = 5). PBMCs were aliquoted into wells at 6 × 10^6^ cells/well. After activation, the cells were cultured with antiretrovirals in the presence of individual drugs in DMSO or DMSO alone as control. All conditions were tested in the presence of CD3/28 T cell–activating beads, except for the unactivated DMSO condition. After 6 days, total cellular RNA was extracted, and the progression through different stages of HIV transcription was quantified by measuring the levels of initiated (TAR), 5′ elongated (LLTR), midtranscribed/unspliced (Pol), completed (PolyA), and multiply spliced (TatRev) HIV transcripts. (**A**) The levels of all HIV transcripts were normalized to 1 μg of total cellular RNA and expressed as a percentage of the activated DMSO control (% of activated DMSO). Medians are shown as well as the individual values per study participant in different colors. (**B**–**D**) The effect of each drug on HIV transcriptional progression after activation was analyzed by the ratio of one HIV transcript to another. Ratios are independent of HIV infection frequency or normalization to cell numbers. Shown are the proportion of (**B**) all HIV transcripts that are elongated (LLTR/TAR), (**C**) elongated HIV transcripts that are completed (PolyA/LLTR), and (**D**) completed transcripts that are multiply spliced (TatRev/PolyA). Medians and IQR are presented, along with the individual values per study participant in different colors. *P* values were calculated using Wilcoxon’s signed-rank test: *P < 0.05.

Next, we evaluated the ability of the candidate LPAs to sustain the inhibitory effects during a prolonged ex vivo culture. To this end, PBMCs were cultured in the presence of individual drugs in DMSO or in DMSO alone as control, activated (anti-CD3/CD28), and harvested after 6 days. None of the drugs significantly reduced viability compared to activated DMSO (Supplemental Figure 4A). Ruxolitinib and triptolide caused sustained reductions in initiated HIV transcripts (medians relative to activated DMSO: 27%, *P* = NS and 1%, *P* = 0.03, respectively; Figure 4A and Table 3). Mithramycin A was not selected from our drug screen initially due to the lack of an effect at 24 hours (Figure 1B and Supplemental Figure 2G). However, we decided to test the drug on day 6 because extra cells were available from some study participants.

Although dinaciclib, tanespimycin, and mithramycin A showed no effects on transcriptional initiation after 24 hours (Figure 3 and Table 2), they reduced initiated HIV transcripts after 6 days (48%, 15%, and 13%, respectively; all *P* = 0.02; Figure 4A and Table 3). Compared with 24 hours, tanespimycin induced greater reductions in completed and multiply spliced HIV transcripts at day 6 (3% and 0%, respectively; both *P* = 0.02). On day 6, the CDK inhibitors alvocidib, dinaciclib, and AZD4573 reduced elongated HIV transcripts (Figure 4A and Table 3; median relative to activated DMSO between 50% and 72%; all *P* = 0.02, except *P* = 0.06 for AZD4573), but the reduction was less than at 24 hours (Figure 3A and Table 2; median relative to activated DMSO at 24 hours between 25% and 37%). The CDK inhibitors also reduced completed and multiply spliced HIV RNA at day 6, albeit to a lesser extent than the reductions observed after 24 hours. The effects of quercetin on midtranscribed and multiply spliced HIV RNA at 24 hours were no longer observed at day 6.

In contrast with 24 hours, the splicing inhibitors had little impact on elongated HIV RNA at day 6, but the effect on multiply spliced HIV RNA was partially sustained (23%, 0%, and 3% for pladienolide B, isoginkgetin, and herboxidiene, respectively; *P* = 0.03 for isoginkgetin; Figure 4A and Table 3). Compared with 24 hours, KRIBB11 tended to show less effect on elongated (72%) and multiply spliced HIV RNA (38%). After correcting for multiple comparisons, we only obtained significant reductions in 5′ elongated HIV transcripts for ruxolitinib, alvocidib, dinaciclib, tanespimycin, and mithramycin A (Table 3 and Supplemental Data 1), indicating a reduced ability of the drugs to maintain their effectiveness until day 6 compared with the initial 24 hours. In general, we observed greater variability in the effect of the drugs among study participants after 6 days (Supplemental Figure 4, B–F). Some drugs had no effect or showed nonsignificant increases in the levels of different HIV transcripts compared to activated DMSO in certain study participants, which was very rare after 24 hours (Supplemental Figure 3, A–E).

When calculating the ratio of one RNA to another on day 6, we observed little effect of the drugs on HIV transcriptional elongation and completion (Figure 4, B and C). However, the ratio of multiply spliced transcripts to completed HIV RNA (TatRev/PolyA) was significantly reduced for both alvocidib (*P* = 0.02) and isoginkgetin (*P* = 0.03; Figure 4D), suggesting a sustained inhibition of those drugs on HIV splicing. After correcting for multiple testing, these *P* values were no longer significant. However, the power was limited because of some participants who had undetectable TatRev or PolyA transcripts, resulting in values of 0 or not defined (0/0 or *x*/0), especially for tanespimycin (2 out of 7) and triptolide (5 out of 6).

### CDK inhibitors appear to decrease baseline HIV splicing.

For a selection of the compounds, we evaluated the ability to reduce baseline HIV transcription without activation. PBMCs were cultured in the presence of individual drugs in DMSO or DMSO alone as a control and harvested after 24 hours. For each drug, we tested PBMCs from at least 3 PWH, as indicated in Supplemental Figure 5B. In line with previous studies (33), ruxolitinib and KRIBB11 (34) had little effect on the HIV transcription levels in the absence of T cell activation (Supplemental Figure 5, A and B). The CDK inhibitors dinaciclib and AZD4573 did not reduce the levels of 5′ elongated or completed HIV transcripts compared to DMSO, but they eliminated multiply spliced HIV transcripts in all study participants (Supplemental Figure 5C; median relative to DMSO: both 0%). Likewise, pladienolide B appeared to reduce the level of multiply spliced transcripts (33%, Supplemental Figure 5, A and B).

When evaluating the ratio of one RNA transcript to another (Supplemental Figure 5D), all tested drugs seemed to cause a modest reduction in HIV transcriptional elongation (LLTR/TAR). However, dinaciclib, AZD4573, and pladienolide B appeared to severely reduce HIV splicing (TatRev/PolyA). Due to the limited number of participants tested, these effects did not reach statistical significance.

### Several LPAs block the production of supernatant viral RNA after T cell activation.

**Figure 5:**
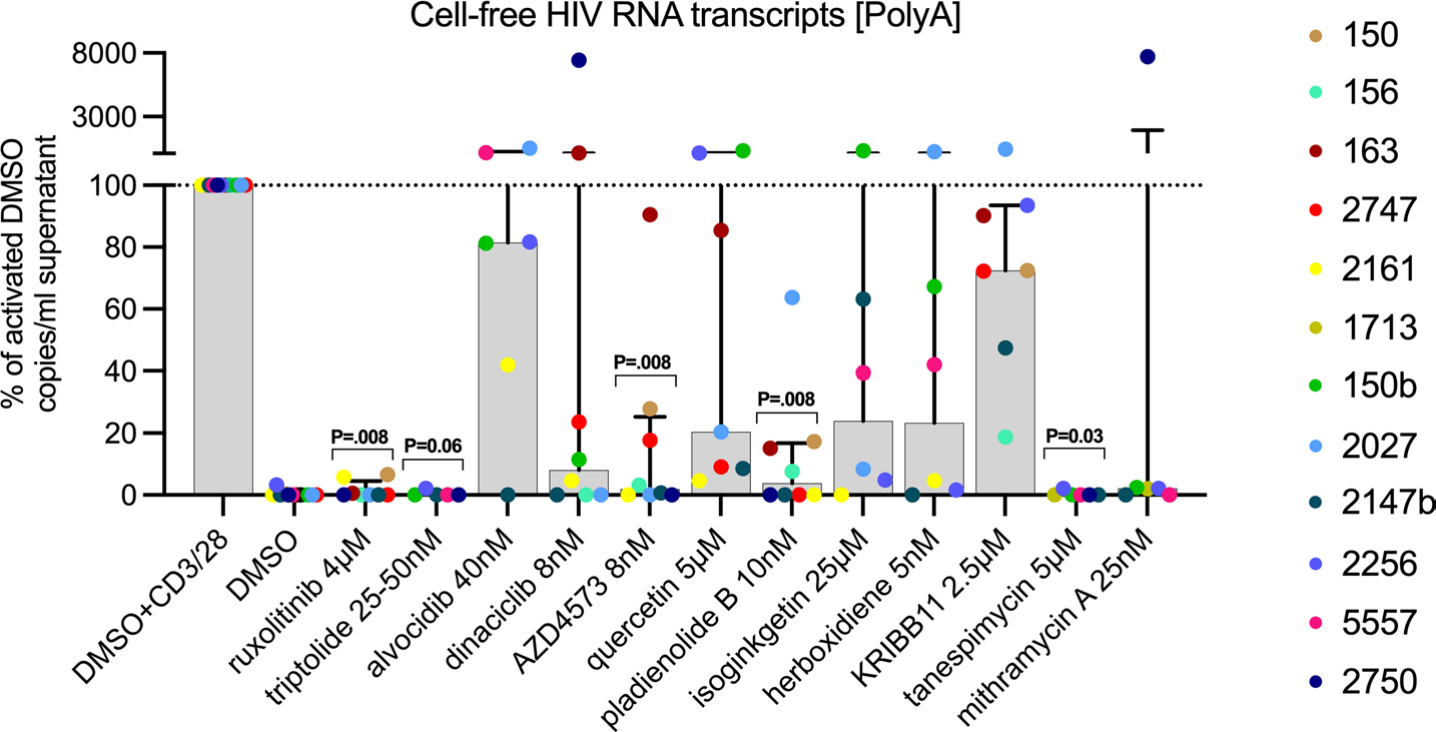
Several LPAs block the production of viral RNA in supernatant after T cell activation. Each drug was tested in PBMCs from 6 ART-suppressed study participants (except for triptolide, *n* = 5). PBMCs were aliquoted into wells at 6 × 10^6^ cells/well. After activation, the cells were cultured with antiretrovirals in the presence of individual drugs in DMSO or DMSO alone as control. All conditions were tested in the presence of CD3/28 T cell–activating beads, except for the unactivated DMSO condition. After 6 days, RNA was extracted from the culture supernatant. Polyadenylated HIV RNA in the supernatant was quantified by RT-ddPCR, expressed as copies/mL, and then normalized to the levels of the activated DMSO (% of activated DMSO). Medians and IQR are presented, as well as the individual values per study participant in different colors. Comparisons were performed using Wilcoxon’s signed-rank test.

Next, we evaluated if the drugs were able to limit the production of supernatant viral RNA after stimulation. PBMCs were cultured in the presence of individual drugs in DMSO or DMSO alone as control, activated (anti-CD3/CD28), and assessed for levels of PolyA HIV RNA in the supernatant at day 6. Ruxolitinib, triptolide, and tanespimycin inhibited the release of viral RNA in supernatant almost entirely (Figure 5; median relative to activated DMSO: all 0.0%; *P* = 0.008, *P* = 0.06, and *P* = 0.03, respectively). The CDK inhibitor AZD4573 and the splicing inhibitor pladienolide B also reduced the production of viral RNA in the supernatant (1.9% and 3.8%, respectively; *P* = 0.008 for both). Ruxolitinib, AZD4573, and pladienolide B significantly reduced viral RNA production in supernatant even after correcting for multiple comparisons (Supplemental Data 1). Dinaciclib, quercetin, isoginkgetin, herboxidiene, and mithramycin A also tended to reduce the median levels of supernatant HIV RNA, but the effects were not consistent enough to reach statistical significance.

### Lower levels of HIV transcripts are not attributed to reduced infection frequency.

**Figure 6:**
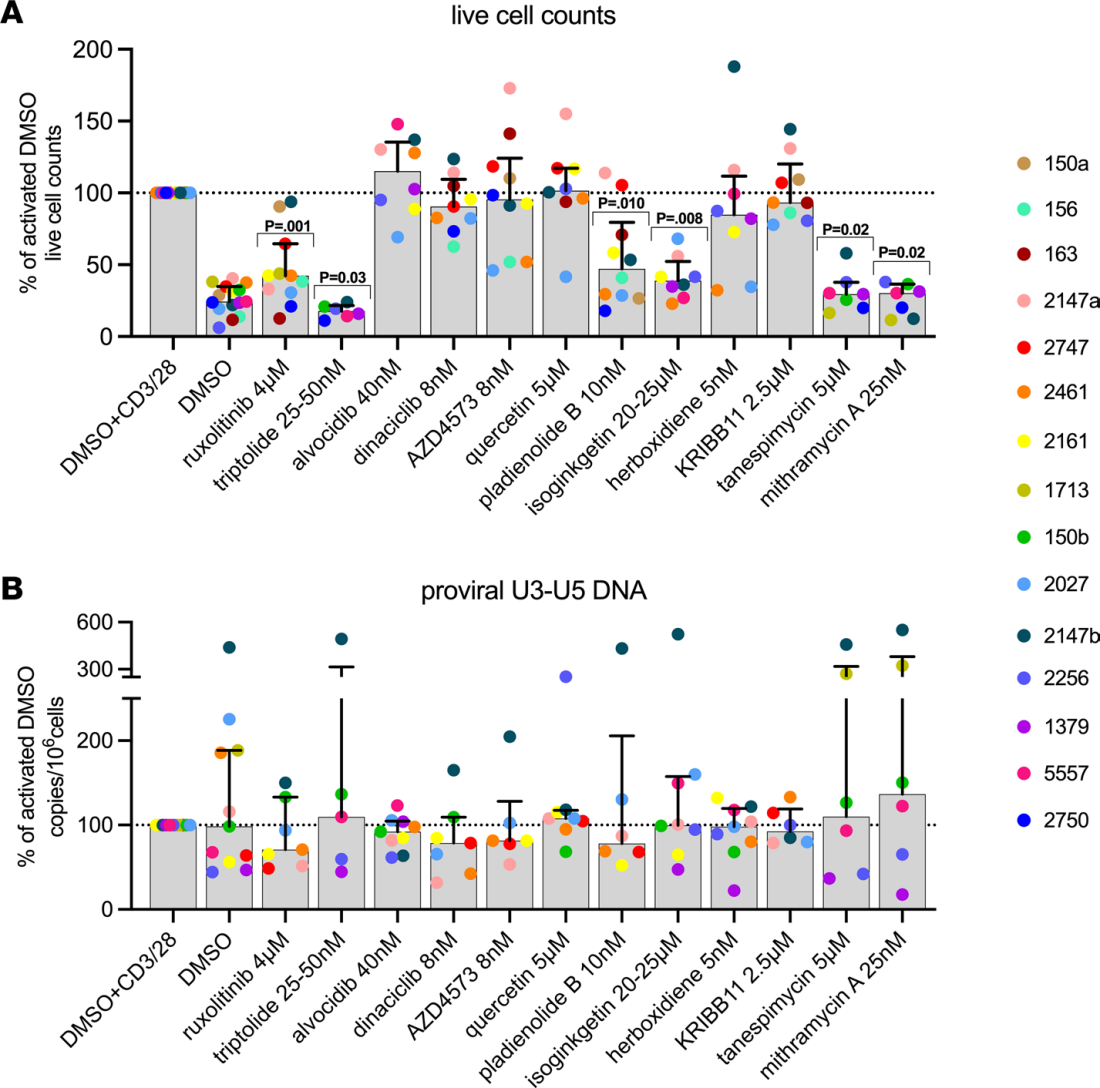
Some LPAs block cell proliferation but do not affect infection frequency. (**A**) After 6 days, the total number of live cells was measured by trypan blue staining and then normalized to the levels of the activated DMSO (% of activated DMSO). All conditions were tested in the presence of CD3^+^/CD28^+^ T cell–activating beads, except for the unactivated DMSO condition. Bars indicate medians, and different colors indicate individual study participants. Comparisons were performed using Wilcoxon’s signed-rank test. (**B**) Total cellular DNA was extracted, and the levels of U3-U5 HIV DNA were measured to quantify infection frequency at 6 days. HIV DNA levels were normalized to copies per 10^6^ cells using the input of cellular DNA (assuming 1 μg of total DNA corresponds to 160,000 cells) and then normalized to the activated DMSO control (% of activated DMSO). Bars indicate medians, and different colors indicate individual study participants.

Some of our candidate LPAs can block the effects of T cell activation, which could affect T cell proliferation. Therefore, we evaluated the extent to which variations in the level of infected cells may contribute to reductions in different HIV RNA transcripts. We measured the total number of viable cells (as a measure of proliferation) and the conserved U3-U5 LTR HIV DNA region (as a measure of total infection frequency) at day 6. Total live cell numbers were significantly lower for PBMCs treated with ruxolitinib, pladienolide B, isoginkgetin, tanespimycin, mithramycin A, and triptolide compared with the activated DMSO control, indicating reduced proliferation after T cell stimulation (all *P* ≤ 0.03; Figure 6A). In contrast, CDK inhibitors, quercetin, herboxidiene, and KRIBB11 did not change proliferation rates compared to the activated DMSO control. Despite differences in cell proliferation, we found no significant differences in total LTR HIV DNA after treatment with any of the drugs (Figure 6B), indicating that differences in cell proliferation rates have no impact on infection frequency (median infection frequency among study participants was 600 HIV copies/~10^6^ cells, or 0.06%, at day 6). This result implies that the observed decreases in HIV transcripts are not attributed to reduced proliferation or killing of infected cells but instead due to enhancement of blocks at the different stages of HIV transcription.

## Discussion

The goals of this study were to discover new LPAs that reduce or prevent HIV expression after T cell activation, compare them with drugs previously reported to reduce HIV transcription, and investigate the mechanisms underlying HIV latency/reactivation. We screened 26 small molecules, of which most are being tested in human trials or are FDA approved (mostly for the treatment of cancer; Supplemental Table 1). Our study demonstrated that certain drugs inhibit specific stages of HIV transcription in cells obtained from PWH ex vivo without impacting cellular viability. We identified new candidate LPAs, including CDK inhibitors (dinaciclib and AZD4573) and splicing inhibitors (pladienolide B, isoginkgetin, and herboxidiene). Some drugs (CDK inhibitors, splicing inhibitors, tanespimycin, and triptolide) inhibited multiple stages of HIV transcription (schematic overview in Supplemental Figure 1) and blocked the production of supernatant viral RNA. Additionally, the CDK inhibitors (dinaciclib and AZD4573) and pladienolide B appeared to inhibit the baseline expression of multiply spliced HIV transcripts in unstimulated PBMCs (Supplemental Figure 5).

HIV transcriptional 5′ elongation and splicing were most sensitive for HIV suppression at 6 days following drug exposure. The CDK inhibitors and mithramycin A durably reduced 5′ elongation for 6 days. The splicing inhibitors, in contrast, did not sustain the inhibitory effects on 5′ elongation after a 6-day culture, but the effect on splicing was partially sustained (Figure 4). It should be noted that we had less statistical power at day 6 compared with 24 hours, because of a smaller number of individuals tested per drug, and that there was an amplification failure of Pol and TatRev transcripts in participant 2461. Of note, there was also an amplification failure of TatRev in participant 2027 at 24 hours. In addition, it is possible that some drugs degraded over 6 days in the cell culture medium, since we did not add fresh drugs after day 0.

None of the tested drugs increased the infection frequency (Figure 6B), which is promising since we aim to avoid expanding the reservoir, but at the same time, none of the drugs reduced the infection frequency. Previous studies have shown that ex vivo treatment of CD4^+^ T cells from viremic individuals with ruxolitinib decreased the frequencies of infected cells with integrated HIV (21). The effect was attributed to reduced antiapoptotic Bcl-2 expression, a downstream target of STAT5 signaling. The absence of this effect in the current study could be attributed to the differential expression of antiapoptotic genes between viremic and ART-suppressed study participants (37). We also used PBMCs, which are a more representative cell type than CD4^+^ T cells but have lower infection frequencies and could also show the effects of other cell types (for example, CD8^+^ T cells) on HIV transcription.

### Triptolide.

The most dramatic inhibition of HIV transcription was observed after treatment with triptolide, with a more than 95% decrease in initiated TAR transcripts. Triptolide is a general RNA polymerase inhibitor (38, 39), induces proteasomal degradation of Tat (40), and inhibits NF-κB signaling (41). Furthermore, triptolide has been investigated to modulate cancer gene expression via epigenetic downregulation of genes associated with super-enhancers (e.g., BRD4, MYC, RNA Pol II) (42). Although both initiated (TAR) and 5′ elongated (LLTR) transcripts were decreased relative to activated DMSO, the high ratio of LLTR/TAR transcripts (Figure 3B) indicates that the predominant mechanism is likely through inhibition of cellular (or perhaps Tat-mediated) HIV transcriptional initiation, or epigenetic mechanisms that prevent initiation of HIV transcription, and not at the level of Tat-mediated transcriptional elongation. Although promising, triptolide has limitations regarding bioavailability and toxicity, limiting its clinical potential (43). However, there is promise in the analog LLDT-8, which has shown less toxicity (43, 44).

### CDK inhibitors.

Our data suggest that CDKs (including CDK9) or their downstream targets appear to be involved in activation-induced reversal of the baseline block to HIV transcriptional completion and multiple splicing. In addition, CDKs may contribute to the baseline block to HIV splicing in unstimulated cells. Only a few studies have investigated the effect of CDK inhibitors on HIV expression in cells from PWH. One study showed a potent reduction of HIV expression in cell lines after treatment with FIT-039 (45). However, FIT-039 had no effect on in vitro–infected primary cells. In addition, the CDK inhibitor flavopiridol (alvocidib) was shown to reduce HIV RNA in supernatant from infected cells from PWH even after drug withdrawal (46). Recently, the selective CDK9 inhibitor LDC000067 has been shown to reduce HIV expression in cell lines (47). LDC000067 also reduced multiply spliced TatRev transcripts in cells from PWH, which is congruent with our findings using other CDK inhibitors (Figure 3A), though alvocidib, dinaciclib, and AZD4573 reduced the levels of multiply spliced HIV transcripts more profoundly than LDC000067 in cells from PWH. The same study showed that LDC000067 maintained decreased levels of HIV expression after drug removal in vitro but only in combination with an inhibitor of CDK8/19 (47). A limitation of the current study is that we did not investigate the effects after drug removal.

### Splicing inhibitors.

Control of HIV splicing is vital for HIV replication, as it allows the expression of different mRNAs and proteins at particular stages in the viral life cycle. Previously, we have shown that blocks to HIV splicing represent a conserved mechanism of HIV latency in multiple primary cell models using infectious viruses (48), as well as cells from blood and tissues of HIV-suppressed PWH (8, 10). In addition, we have also identified human splice factors that are differentially expressed upon activation (48). Consequently, interfering with HIV splicing represents an interesting drug target to silence HIV expression. To our knowledge, this is the first time that the splicing inhibitors pladienolide B, isoginkgetin, or herboxidiene have been investigated for their effects on HIV transcription and reactivation. We expected most of the effect to occur on the level of multiply spliced transcripts, as was previously observed for other splicing inhibitors (49). However, pladienolide B and herboxidiene also greatly reduced 5′ elongated HIV transcripts at 24 hours, while isoginkgetin reduced midtranscribed/unspliced HIV transcripts. Moreover, pladienolide B and isoginkgetin caused additional reductions in completed HIV transcripts and reduced the ratio of completed to 5′ elongated HIV transcripts at 24 hours. Our findings indicate that the human splicing factors (such as SF3b1) and/or their downstream targets may be involved in activation-induced reversal of the baseline block to HIV transcriptional elongation, completion, and splicing.

These inhibitors probably reduce the splicing of multiple human transcripts, some of which may encode proteins that normally promote various stages of HIV transcription. Pladienolide B and herboxidiene inhibit the splicing factor SF3b1, which is also known to interact with HIV Tat and the PTEF-b complex (50). As a result, inhibition of SF3b1 reduces RNA Pol II associated with HIV-1 promoter and elongation sites. In line with our data, inhibition of SF3b1 has been associated with reductions in both unspliced and multiply spliced HIV RNA (50), indicating that splicing inhibitors profoundly inhibit HIV expression at multiple stages of HIV transcription.

### Quercetin.

There is conflicting evidence on whether the flavonoid quercetin activates (51) or suppresses (52) HIV gene expression, which may be the result of its multimodal mechanism of action. Quercetin enhances the function of SIRT1 deacetylase, which is responsible for the deactivation of Tat and other factors that induce HIV transcription in T cells (e.g., NF-κB) (53). Second, quercetin inhibits the PI3K/Akt pathway, which favors HIV latency but also HIV reactivation (54). In this study, we observed a gradual decrease in HIV transcriptional progression after 24 hours’ treatment. Unfortunately, this effect was not sustained after 6 days of culture, which may be due to the rapid oxidation of quercetin in the cell culture medium (55).

### HSP90 inhibition by tanespimycin.

Our data illustrate that HSP90 inhibition is a promising pathway to block HIV expression and reactivation. HSP90 localizes to the HIV LTR and upregulates NF-κB, NFAT, and STAT5-induced gene expression (56). In our study, the HSP90 inhibitor tanespimycin emerged as one of the most promising LPAs. Tanespimycin reduced HIV transcriptional elongation at 24 hours and reduced initiated HIV transcripts after 6 days of culture. Second, tanespimycin completely blocked the production of supernatant viral RNA after T cell activation in all 6 study participants tested. Its potential has been demonstrated previously in a study where tanespimycin durably prevented viral rebound in vivo in a humanized mouse model, even after removal of the drug (20). In our study, we showed that tanespimycin potently blocks HIV expression and reactivation in cells from PWH and that the inhibition mainly occurs at the level of HIV transcriptional initiation and elongation, consistent with its proposed mechanism (56).

### The effect of some but not all previously studied LPAs was verified in PBMCs from PWH.

Digoxin and 8-azaguanine have been reported to interfere with the Rev-mediated export of unspliced and single-spliced HIV mRNAs (57), leading to oversplicing and an abundance of multiply spliced HIV transcripts relative to single-spliced and unspliced transcripts. We observed an increase in multiply spliced transcripts after 8-azaguanine but not after digoxin treatment (Figure 1B and Supplemental Figure 2F), perhaps because our digoxin concentration (which was chosen based on therapeutic plasma levels) was lower than that used previously. Moreover, we did not observe decreases in elongated, unspliced, or completed HIV transcripts with either digoxin or 8-azaguanine (Supplemental Figure 2F).

Spironolactone has been reported to inhibit HIV transcription via TFIIH inhibition (58, 59). In our study, we observed little reduction in HIV transcription with spironolactone, perhaps because we used a lower concentration (chosen based on plasma levels in humans). Other differences in the methods may also contribute to discrepancies from prior studies using 8-azaguanine, digoxin, and spironolactone. However, given that these drugs showed little effect on HIV transcription in the first 2 study participants (Figure 1B), we did not pursue further studies with these drugs.

In agreement with other studies, ruxolitinib (21, 22) and triptolide (40, 44, 60) profoundly reduced HIV transcription and reactivation. Although KRIBB11 significantly decreased elongated HIV transcripts at 24 hours (Table 2), this effect was not maintained after 6 days, and KRIBB11 did not inhibit the production of viral RNA in the supernatant. It has been shown that the ability of KRIBB11 to prevent latency reversal depends on the type of reactivating agent (34). In contrast with other studies, we did not observe much reduction in our initial drug screen for aspirin (61) or 53425191 (Figure 1B and Supplemental Figure 2, B and H) (62). For 53425191, which has been shown to alter HIV splicing (62), we did observe a strong reduction in multiply spliced HIV transcripts in cells from 2 study participants at day 6 (Supplemental Figure 6A) but only a 50% reduction in viral RNA in the supernatant (Supplemental Figure 6B). Likewise, the SP-1 inhibitor mithramycin A showed no effect after 24 hours but strongly reduced initiated HIV transcripts after 6 days (Figure 4A). Furthermore, mithramycin A inhibited the production of viral RNA in supernatant in 5 out of 6 study participants (Figure 5), corroborating a prior study in which it reduced latency reactivation (63).

Mesalamine, cordycepin, SPV106, and C26 have not been previously studied for their effects on HIV transcription. The compound C26 was reported to induce cancer cell dormancy by increasing NR2F1 activity (64). Since NR2F1 recruitment by the chromatin remodeling factor RBBP4 was shown to repress LTR-mediated HIV transcription (65) and RBBP4 was shown to be differentially expressed between transcriptionally active and silent proviruses in cells from PWH (66), we tested C26 in this study. However, C26 had no impact on HIV transcription at 24 hours (Figure 1B and Supplemental Figure 2H), nor did it drastically inhibit HIV transcription or the production of viral RNA in supernatant after 6 days in 2 tested participants (Supplemental Figure 6, A and B). Similarly, for mesalamine, cordycepin, and SPV106, we did not observe any reduction in different HIV transcripts after 24 hours compared to activated DMSO (Figure 1B and Supplemental Figure 2, B, F, and I).

### Transcriptional silencing as a strategy to block immune activation.

Chronic immune activation in PWH on ART is linked to non-AIDS morbidities, such as cardiovascular disease, neurocognitive impairment, type 2 diabetes, and cancer (67). The most obvious causes of immune activation are the innate and adaptive immune responses against the virus and its antigens (67). While ART suppresses viral replication to undetectable levels in most PWH, we and others have detected cell-associated HIV RNA in the vast majority of PWH on prolonged ART (8). A considerable portion of these HIV RNAs may be transcribed from defective proviruses; nonetheless, some may activate intracellular defenses or express viral proteins (68–70). Recent evidence shows that those sequences contribute as much, if not more, to immune activation and inflammation in PWH on ART. Both innate and adaptive immunity have been shown to be driven by the expression of mostly defective HIV RNAs and proteins (71, 72). Moreover, multiple studies found no correlation between immune activation or inflammation and the level of intact proviruses (73, 74). Therefore, HIV studies and interventions aimed at reducing immune activation will have to consider both intact and defective proviruses, or at least those that are transcriptionally and translationally active and/or inducible. An advantage of HIV transcription inhibitors as antiviral or cure strategy is their capacity to target all infected cells, without requiring the provirus to be intact or replication competent. Furthermore, transcriptional inhibition can be obtained across different types of infected cells and does not require an immune response. It is still unclear if LPAs will need to be administered as a onetime treatment, intermittently, or for life. The answer to this question depends on the degree to which the effects on HIV transcription are sustained, which will require testing the drugs over longer time spans, potentially with washout periods. Nonetheless, transcriptional inhibitors, even if used as permanent adjuvant to ART, might be beneficial to suppress the residual HIV pathogenesis caused by HIV expression from intact and defective proviruses in PWH.

### Transcriptional silencing in a block-and-lock cure strategy.

The most clinical benefit will likely be obtained from drugs that not only impair HIV transcription but also block the production of viral RNA in supernatant after T cell activation. We observed this effect with ruxolitinib, triptolide, AZD4573, pladienolide B, and tanespimycin. Each of those drugs works by a different mechanism, suggesting the potential for single or combination therapies to prevent reactivation of latent HIV as a proof of concept for a block-and-lock approach to a functional cure. Ruxolitinib has shown promise in clinical trials in PWH for its ability to decrease specific markers of inflammation and immune activation (75). However, no reduction was observed in cell-associated HIV RNA, indicating that the immunomodulatory effect of ruxolitinib in vivo may not be related to reducing HIV transcription. To our knowledge, only 1 drug (ABX464, an antiinflammatory agent and putative Rev inhibitor) has been shown to reduce HIV transcription in ART-suppressed PWH, and the effect was reversed after withdrawing ABX464 (76). One explanation is that many LPAs can inhibit HIV transcription (block), but not all of them can permanently put the provirus in a deep latent state (lock) (24). The determinants of durable HIV suppression remain unclear, but epigenetics may play a major role (reviewed in ref. 77). LPAs eliciting a successful block and lock often induce altered chromatin organization (22, 47, 78–81).

In addition, while there are multiple host pathways to target for HIV silencing, inhibiting these cellular factors/pathways may lead to off-target effects. Our data suggest some specificity of the drugs to block HIV transcription, since all HIV transcripts were normalized to 1 μg of total cellular RNA to account for the effect of the drug on global cellular transcription before further normalization to the DMSO control. Targeting the host transcription or splicing machinery may induce cellular toxicity by interfering with the expression of essential cellular genes. However, HIV transcription may be more dependent on particular cellular genes, while cellular toxicity may be avoided by redundant mechanisms. For example, blocking the expression of certain SR proteins needed for splicing can be compensated by other pathways in the host cell without a significant loss of function but not for HIV splicing (82). To best approximate clinical practice, we selected low drug concentrations, and if known, concentrations that are currently tested in clinical trials for other indications. We did not observe reduced cell viability, which may indicate that clinically acceptable concentrations may not induce cellular off-target effects.

In summary, we have validated existing drugs but also identified new drugs and druggable targets to inhibit HIV transcription and/or latency reactivation ex vivo. Our study gives new insights into the cellular factors governing HIV expression: 1) CDKs (including CDK9) or their downstream targets may contribute to the baseline block to HIV splicing in unstimulated cells; 2) in addition to CDKs, the targets of SF3b1 seem to be involved in activation-induced reversal of the baseline block to HIV transcriptional completion; and 3) in addition to SF3b1, CDKs or their downstream targets appear to be involved in activation-induced reversal of the baseline block to HIV multiple splicing. Targeting HIV transcription as part of ART may provide virological benefits for individuals with drug-resistant HIV strains or nonsuppressible viremia (83). The effects of cell-associated HIV RNA on chronic immune activation and inflammation in suppressed PWH are not well studied. Therefore, future studies should examine the effects of various HIV transcription inhibitors, alone or in combination, on HIV expression, immune activation, and rebound after stopping ART.

## Methods

### Sex as a biological variable.

Deidentified blood samples were supplied by our collaborators from study participants recruited from UCSF’s Scope/Options Cohort or the San Francisco VA Medical Center. The demographics of the study participants reflect those of people living with HIV who are enrolled in Scope/Options or receive care at the San Francisco VA. Given the demographics of the study participants, we were not able to explore sex as a biological variable.

### Cell culture and treatments.

PBMCs were isolated from fresh venous blood from 16 ART-suppressed HIV-infected study participants using Ficoll density gradient centrifugation. The cells were seeded at 6 × 10^6^ cells/well and cultured with antiretrovirals (nevirapine and indinavir) to prevent new infection. On the next day, PBMCs were activated with anti-CD3/CD28–coated beads (Invitrogen) in the presence of 20 U/mL IL-2 and individual drugs in DMSO or DMSO alone as a control. Since we were limited in the PBMC yield per study participant, different study participants were used for different experiments (such as the initial screen, dose response, different time points, and studies in activated vs. unstimulated cells). In addition, the yields of PBMCs were sometimes insufficient to test all drugs for a given question or experiment.

For quantification of cell-associated HIV transcripts, PBMCs were harvested either after 24 hours or after 6 days. The cell viabilities were measured by trypan blue staining; then the cells were pelleted by centrifuging at 300*g* for 6 minutes and stored at –80°C. For quantification of virion-associated HIV RNA, cell culture supernatant was collected from 6 × 10^6^ PBMCs that were treated and activated for 6 days as described above. Residual cells and cellular debris were subsequently removed from the supernatant by differential centrifugation (300*g* for 6 minutes, then 20,000*g* for 10 minutes), and the supernatant was stored at –80°C.

### Nucleic acid isolation, reverse transcription, and ddPCR.

Total cellular DNA and RNA were extracted in parallel using Trireagent (Molecular Research Center) according to the manufacturer’s instructions, except for study participants 2147 and 2461 (Figure 4), for whom DNA and RNA were extracted using the QIAGEN AllPrep DNA/RNA/miRNA Universal Kit with on-column DNase I treatment of the RNA and the manufacturer’s modification to enhance recovery of short transcripts. RNA and DNA concentrations were measured using UV spectrophotometry (NanoDrop One, Thermo Fisher Scientific). Total initiated HIV (TAR) transcripts were quantified by a 3-step polyadenylation RT-ddPCR as described previously (8, 84). HIV 5′ elongated (LLTR), midtranscribed (Pol), completed (PolyA), and multiply spliced (TatRev) transcripts were quantified by a 2-step RT-ddPCR as previously described (8). The levels of each HIV transcript were normalized to 1 μg of total cellular RNA and expressed as a percentage of the (activated) DMSO control. To quantify virion-associated RNA in supernatant, RNA was extracted from 400 μL supernatant using Trireagent-LS (Molecular Research Center) according to the manufacturer’s instructions. Levels of U3-polyadenylated HIV RNA were measured in the supernatant by RT-ddPCR, normalized to copies/mL, and expressed as a percentage of the activated DMSO. For measuring the HIV infection frequency at day 6, the HIV DNA U3-U5 LTR region was quantified by using ddPCR as described previously (8). HIV DNA copies were normalized to cell numbers according to the mass of DNA input per well (calculated from the DNA concentration and input volume) (85).

### Statistics.

The Wilcoxon signed-rank test (2 tailed) was used to compare the relative measures of different HIV RNA transcripts with the (activated) DMSO. A *P* value less than 0.05 was considered significant. The wells with no positive droplets (observed after LPA treatment) were assigned a value of 0 to calculate the median and *P* values (8, 10). All statistics were performed using GraphPad Prism (Version 9.5.1). *P* values were corrected for multiple comparisons using the Benjamini-Hochberg method as outlined in Supplemental Data 1.

### Study approval.

HIV-infected study participants were recruited sequentially from the San Francisco VA or the UCSF Scope/Options cohort. The study was approved by the local Institutional Review Board of UCSF and the San Francisco Veterans Affairs Medical Center. All participants provided written informed consent.

### Data availability.

Data are available in the Supporting Data Values XLS file or from the corresponding author upon request.

## Author contributions

SAY and JKW designed the study; SGD and JKW provided samples; SAY, PK and JJ designed experiments; JJ, PK, SJK, AW, and GNK conducted experiments; JJ, PK, and SAY analyzed data; JJ and SAY wrote the original draft; all authors reviewed and edited the manuscript; and SAY, JKW, and PWH provided supervision and funding. All authors read and approved the final manuscript.

## Supplementary Material

Supplemental data

Supplemental data set 1

Supporting data values

## Figures and Tables

**Table 1:**
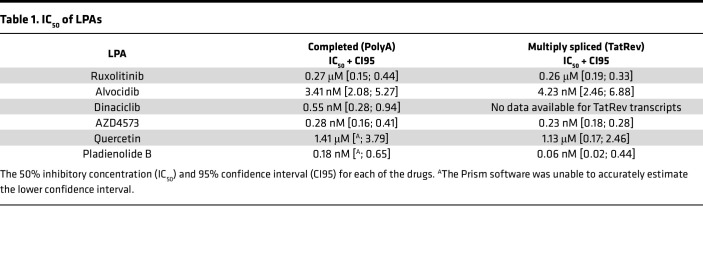
IC_50_ of LPAs

**Table 2:**
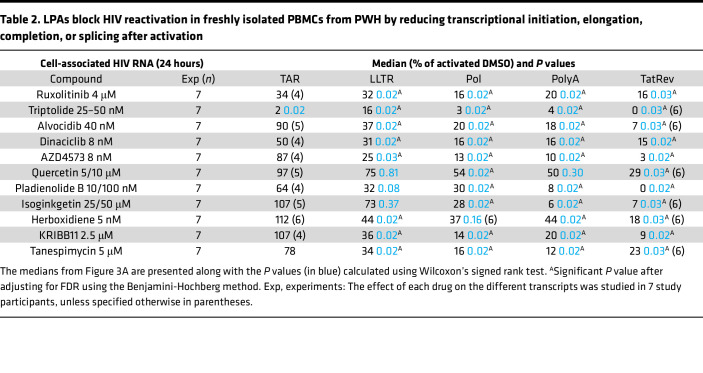
LPAs block HIV reactivation in freshly isolated PBMCs from PWH by reducing transcriptional initiation, elongation, completion, or splicing after activation

**Table 3:**
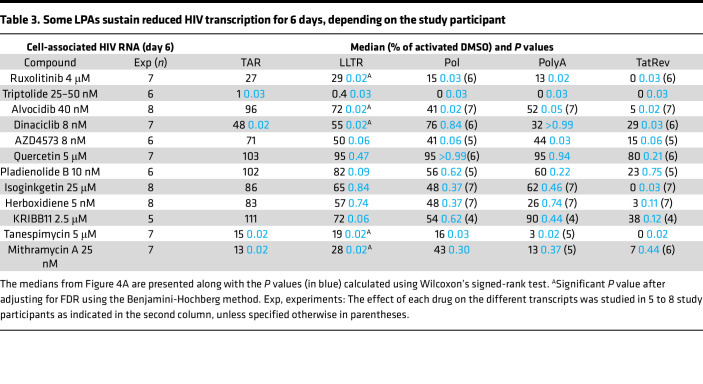
Some LPAs sustain reduced HIV transcription for 6 days, depending on the study participant

## Version 1 08/20/2024

In-Press Preview

## Version 2 10/08/2024

Electronic publication
