# Supplementary material for: Mechanisms and efficacy of small molecule latency-promoting agents to inhibit HIV reactivation ex vivo
Source: JCI Insight. 2024 Aug 20;9(19):e183084. doi: 10.1172/jci.insight.183084 (PMC11466185; doi:10.1172/jci.insight.183084)
Supplement: Supplemental data [file jciinsight-9-183084-s046.pdf]

Supplementary information

Supplementary Table 1. Latency promoting agents: overview of indication, clinical trial status and potential effect on HIV transcription and/or reactivation.

**Compound**	**Target**	**Indication and usage (status)**	**Clinical trials**	**Effect on HIV transcription and/or reactivation**	**Refs**
ruxolitinib	Jak/STAT	Rheumatoid arthritis (RA) and myelofibrosis (FDA approved)	Phase II to study inflammation and immune activation in people with HIV (PWH) on ART	Downregulation of immunomodulatory cytokines (IL6, IL2, TNFalpha) that contribute to chronic inflammation, chronic infection, and HIV disease progression	(1–3)
aspirin	NF-κB inhibitor	Non-steroidal anti-inflammatory drug (FDA approved)	Phase I to reduce cardiovascular disease in ART-treated PWH; tested in combination with HCQ as HIV prevention strategy	Inhibition of NF-κB-dependent HIV transcription	(4)
mesalamine	NF-κB	Inflammatory bowel disease (FDA approved)	Phase I/II to reduce immune activation and inflammation in ART-treated PWH	Inhibition of NF-κB-dependent HIV transcription	
spironolactone	TFIIH inhibitor	Potassium-sparing diuretic for hypertension and cardiovascular disease (FDA approved)	Phase III trial for its anti-inflammatory properties in RA	Inhibition of HIV transcriptional initiation by proteasomal degradation of the XPB subunit of TFIIH	(5, 6)
resveratrol	SIRT-1 activator	Supplement with antioxidant, anti-inflammatory, immunomodulator, lipid regulatory,	Ongoing trials for treatment of several cancers; for its neuroprotective	Inhibition of Tat-dependent HIV transcription via SIRT1 activation	(7)

		and neuroprotective effects	effects and for the treatment of diabetes type 2		
triptolide	Inhibitor of NF-κB, RNA Polymerase (RNA P), and Tat	Used in Chinese traditional medicine for immunosuppressive (RA), anti-inflammatory and anti-cancer properties	Phase I/II/III to reduce immune activation, inflammation, and the HIV reservoir in PWH; for small cell lung cancer; polycystic kidney disease; and Crohn's disease	Proteasomal degradation of Tat; reduction of enhancer mediated transcription by reducing H3K27ac and BRD4 occupancy; Inhibition of NF-κB and NFAT signaling; Inhibitor of RNAP I, II, III	(8–12)
digoxin	vRNA processing inhibitor	Cardiac glycoside used to treat heart failure and atrial fibrillation (FDA approved)	Several trials for various cancers	Inhibition of HIV protein synthesis by inducing oversplicing of the HIV mRNA	(13, 14)
8-azaguanine	vRNA processing inhibitor	Purine analogue	-	Induces oversplicing of HIV mRNA and inhibits Rev function	(15)
alvocidib	CDK1,2,4,9,6 inhibitor	Orphan status for B-CLL, AML	Phase I completed for advanced solid tumors with TP-1287, a prodrug of alvocidib	Inhibition of CDK-induced RNAPII phosphorylation	(16)
dinaciclib	CDK2,5,1,9 inhibitor	-	Phase I/II for solid tumors and melanoma	Inhibition of CDK-induced RNAPII phosphorylation	(16)
AZD4573	Selective CDK9 inhibitor	-	Phase II for T-cell lymphoma, classical Hodgkin's lymphoma and hematologic malignancies	Inhibition of CDK-induced RNAPII phosphorylation	(16)
atuveciclib	PTEF-b/CDK9 inhibitor	-	Phase I for hematologic malignancies (completed)		(16)
staurosporine	PKC a,g,n,d,e inhibitor	-	Phase I/II for 7-hydroxy-staurosorine and midostaurin to	Proapoptotic effects triggered by inhibition of PKC-	(17)

			treat various cancers	induced gene expression	
sotrastaurin	PKC pan-inhibitor	-	Phase II for B-cell lymphoma and uveal melanoma; Phase II for kidney transplant rejection	Inhibition of PKC-induced gene expression inhibits T cell activation	(17)
enzastaurin	PKC b inhibitor	Glioblastoma (FDA orphan status)	Phase I/II/III for various cancers; Phase I for central nervous system malignancies	Inhibition of NF-κB-mediated transcription induces antiproliferative and proapoptotic effects	(17)
quercetin	SIRT1 activator and PI3K inhibitor	Flavonoid known for its antioxidant, antiviral, antimicrobial, and anti-inflammatory, and neuroprotective effects (FDA GRAS status as dietary supplement)	Ongoing trials for its use in inflammatory, cardiovascular, metabolic diseases and aging	SIRT-1 deacetylation reduces Tat activity and downregulates induction factors of T cells: NF-κB, activator protein 1 (AP-1) and IL-2; Inhibition of PI3K/Akt pathway interferes with latency reactivation	(18–21)
SPV-106	HAT inhibitor	-	-	Inhibition of p300-HAT reduces Tat transactivation	(22)
cordycepin	polyA inhibitor	Used in Chinese traditional medicine for its potent anti-cancer, antioxidant and anti-inflammatory effects	Phase I/II for leukemia, solid tumors, and lymphoma	Adenosine analog that functions as chain terminator for mRNA polyadenylation by poly(A) polymerases	(23)
pladienolide B	splicing inhibitor	-	Phase I with synthetic analogues (H3B-8800; E7107) for myelodysplastic syndromes and solid tumors	Inhibits the function of splicing factors SAP130, SF3b1, and U2 snRNP	(24, 25)
isoginkgetin	splicing inhibitor	-	-	Pre-mRNA splicing inhibition by	(26)

				preventing stable recruitment of the U4/U5/U6 tri-small nuclear ribonucleoprotein	
herboxidiene	splicing inhibitor	-	-	Inhibits the function of splicing factor SF3b1 subunit SAP145	(27)
KRIBB11	HSF1/PTEF-B	-	-	Inhibits HSF-1 from recruiting P-TEFb to HSP promotors	(28)
tanespimycin	HSP90 inhibitor	-	Phase II for relapsed lymphomas, solid tumors, and leukemia	HSP90 localizes to the HIV LTR and upregulates NF-κB, NFAT and STAT5 induced gene expression	(29–32)
mithramycin A	SP-1 inhibitor	-	In vitro studies for cervical cancer	Inhibits the function of Sp1 that drives viral gene expression	(33, 34)
C26	NR2F1 agonist	-	In vitro and mice studies to induce dormancy in cancer cell lines	NR2F1 recruitment to HIV promotor activates downstream target genes SOX9, RARβ, and p27 that induce global chromatin repression	(35, 36)
5342191	MAPK/ERK modulator	-	-	Activation of MEK1/2-ERK1/2 signalling reduces HIV expression by interfering with RNA splicing	(37, 38)

Abbreviations: PWH, People with HIV; RA, Rheumatoid arthritis; RNA P, RNA polymerase; HQC, Hydroxychloroquine; B-CLL, B cell chronic lymphocytic leukemia; AML, acute myeloid leukemia.

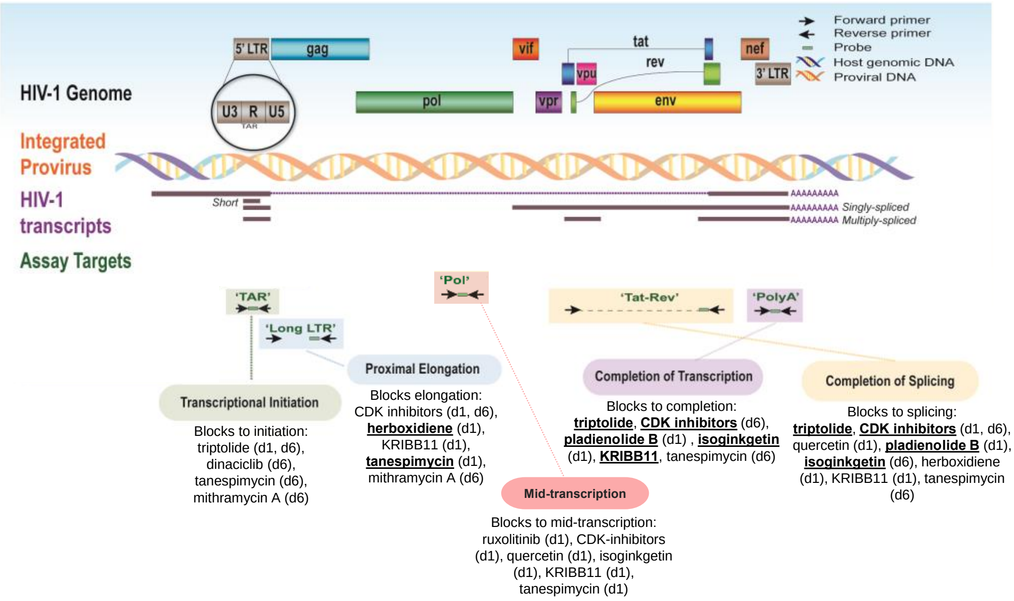

Figure S1. LPAs acting through different mechanisms to induce blocks at various stages of HIV transcription. This schematic shows the genetic organization of proviral HIV DNA and the HIV 'transcription profiling' assays targeting specific RNA sequence regions that provide insight into blocks to HIV transcription. Promising LPAs that induce specific blocks at different steps of transcriptional reactivation are listed. d1 indicates LPAs that significantly reduced the respective HIV transcript relative to activated DMSO at day 1 (24h), while d6 indicates LPAs that significantly reduced the HIV transcript relative to activated DMSO on day 6. Drugs that significantly altered the ratios at day 1 and/or day 6 are shown in bold and are underlined. Figure adapted from (39).

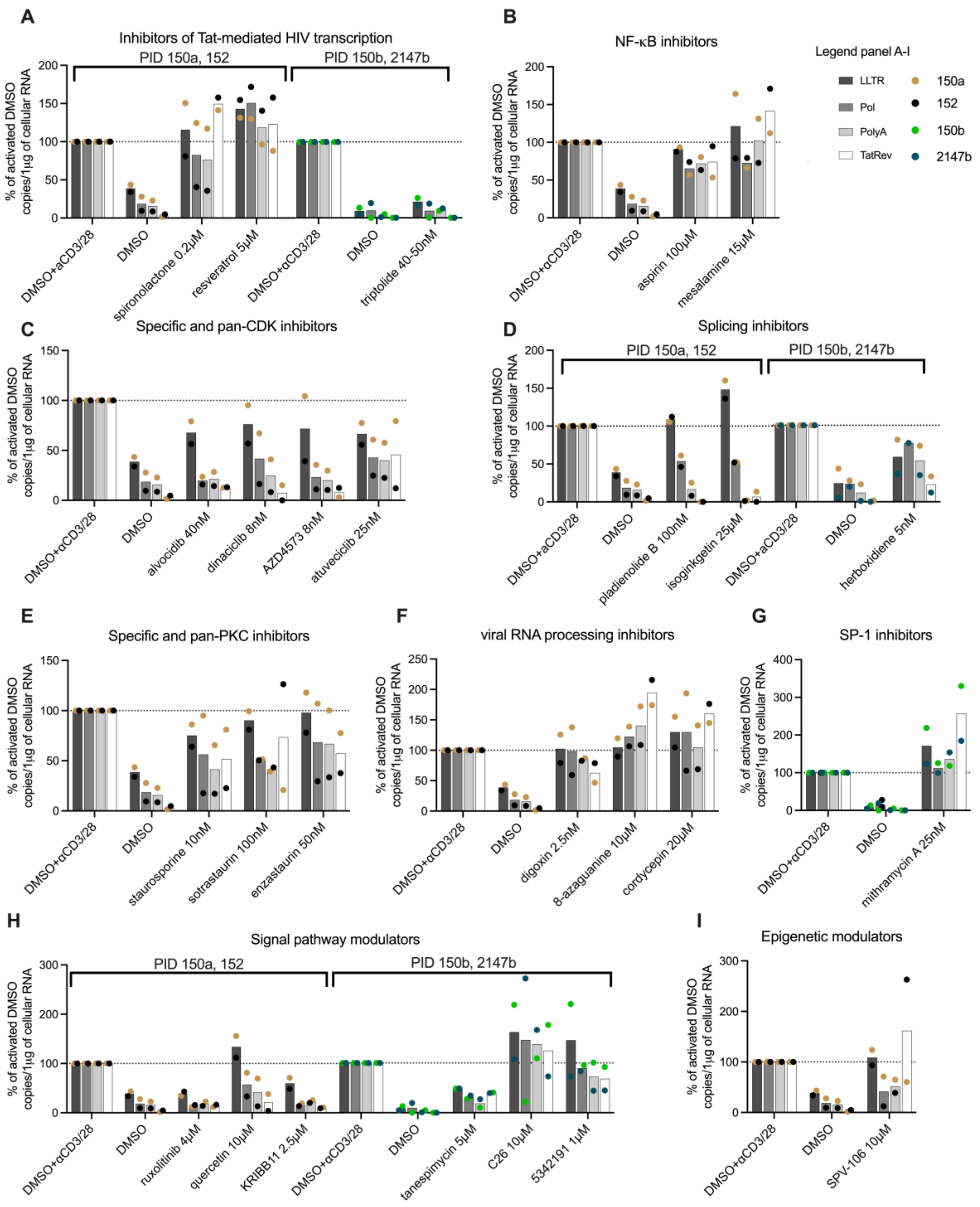

Figure S2. Screening of candidate LPAs by measuring changes in 5' elongated, mid-transcribed, completed, and multiple spliced HIV transcripts after activation. (A-I) Each drug was tested in PBMCs from two ART-suppressed study participants and the PBMCs were aliquoted into wells at 6x10^6 cells/well. After activation, the cells were cultured with antiretrovirals and individual drugs or controls (DMSO and activated DMSO). All drugs

were tested in the presence of CD3/28 T cell activating beads, except for the unactivated ‘DMSO’ condition. Total cellular RNA was extracted after 24h and 5’ elongated (LLTR), Pol (unspliced, mid-transcribed), completed (PolyA), and multiply spliced (TatRev) HIV transcripts were quantified by RT-ddPCR. The levels of each transcript were quantified, normalized to 1 µg of total cellular RNA, and expressed as a percent of the activated DMSO control (% of activated DMSO). Medians are shown, as well as the values of the individual study participants in different colors.

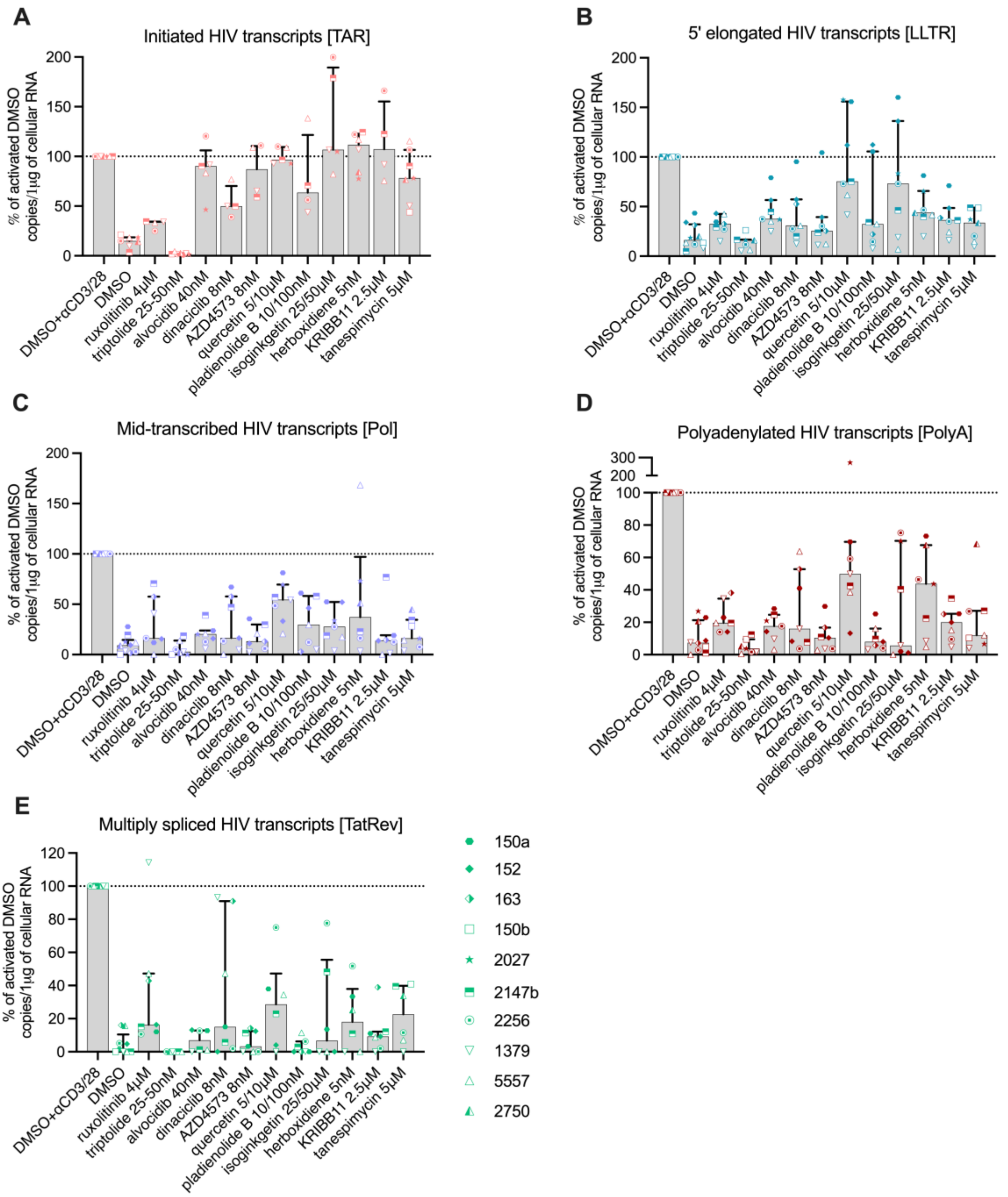

Figure S3. Various LPAs impact distinct phases of HIV transcription upon activation.

A-E) Each drug was tested in PBMCs from seven ART-suppressed study participants and the PBMCs were aliquoted into wells at $6x10^6$ cells/well. After activation, the cells were cultured with antiretrovirals in the presence of individual drugs in DMSO or DMSO alone as control. After 24h, total RNA was extracted and the progression through different stages of HIV transcription was quantified by measuring the levels of **A)** initiated (TAR), **B)** 5’ elongated

(LLTR), **C)** mid-transcribed/unspliced (Pol), **D)** completed (PolyA) and **E)** multiply spliced (TatRev) HIV transcripts. The levels of all HIV transcripts were quantified, normalized to 1 μg of total cellular RNA, and expressed as a percent of the activated DMSO control (% of activated DMSO). Medians with IQR are shown, and varying symbol shapes denote individual study participants.

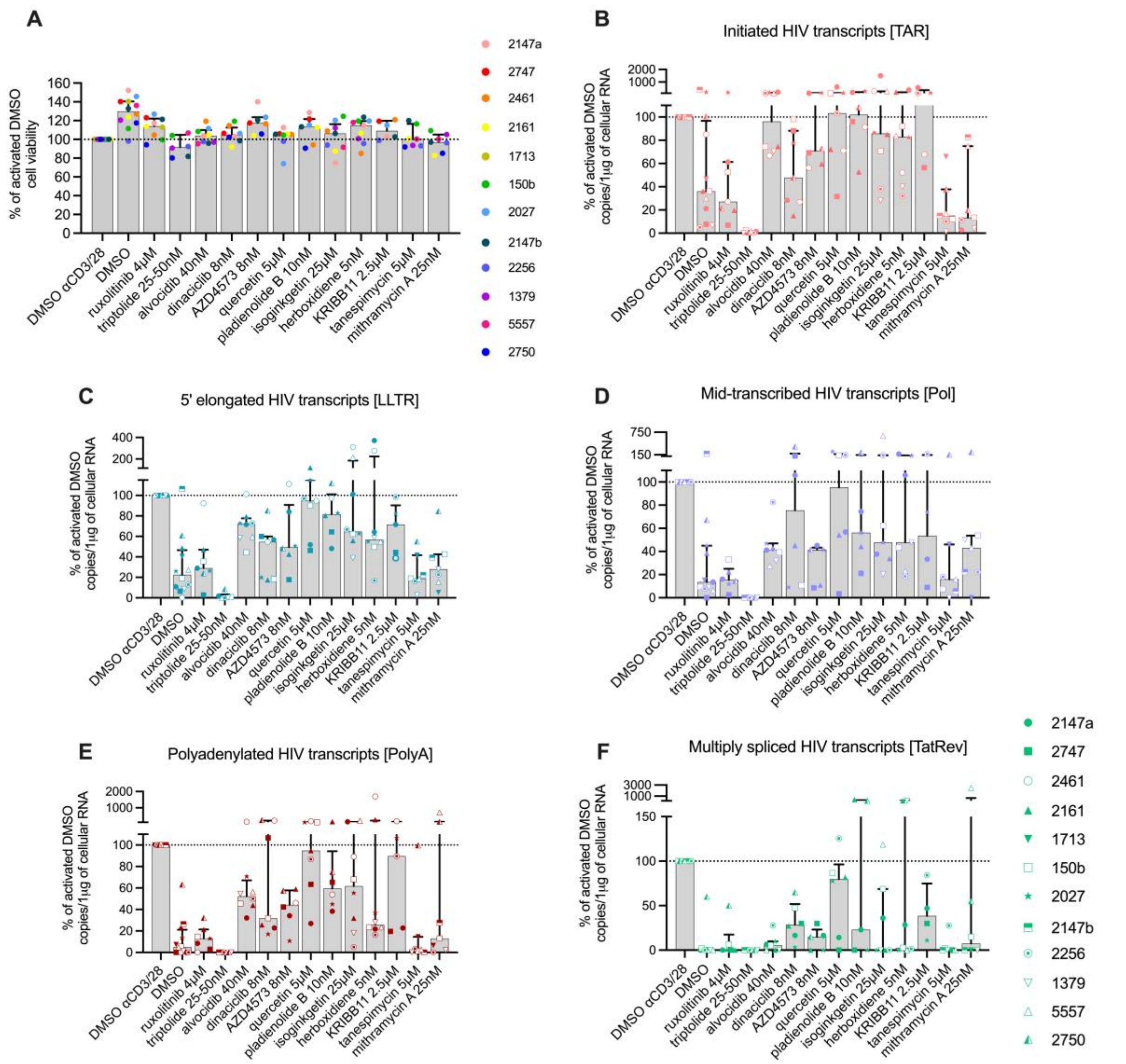

Figure S4. Some LPAs sustain reduced HIV transcription for six days depending on the study participant but without effect on cellular viability. A) After six days, the viability was measured by trypan blue staining and then normalized to the levels of the activated DMSO (% of activated DMSO). Bars indicate medians and different colors indicate individual study participants. **B-F)** Each drug was tested in PBMCs from at least six ART-suppressed study participants (except for KRIBB11, n=5) and the PBMCs were aliquoted into wells at $6x10^6$ cells/well. After activation, the cells were cultured with antiretrovirals in the presence of individual drugs in DMSO or DMSO alone as control. After six days, total RNA was extracted and the progression through different stages of HIV transcription was

quantified by measuring the levels of **B)** initiated (TAR), **C)** 5’ elongated (LLTR), **D)** mid-transcribed/unspliced (Pol), **E)** completed (PolyA) and **F)** multiply spliced (TatRev) HIV transcripts. The levels of all HIV transcripts were quantified, normalized to 1 µg of total cellular RNA, and expressed as a percent of the activated DMSO control (% of activated DMSO). Medians with IQR are shown, and varying symbol shapes denote individual study participants.

A

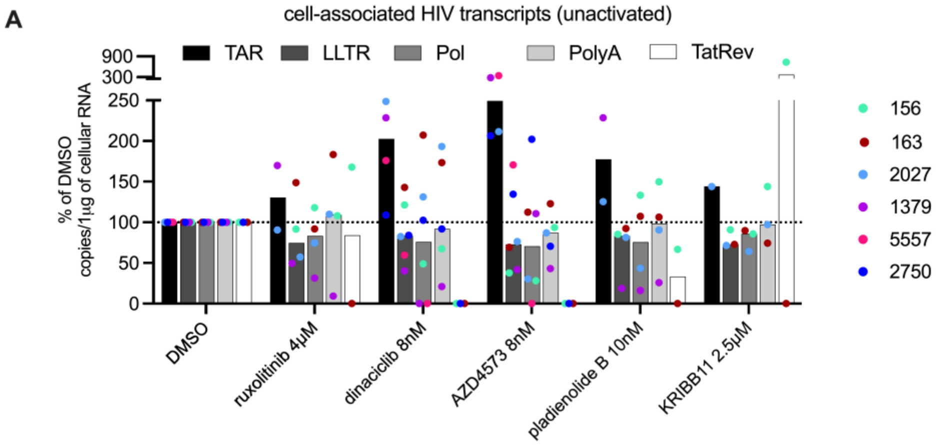

B

cell-associated HIV RNA (24h)		Median (% of DMSO)				
Compound	Exp (n)	TAR	LLTR	Pol	PolyA	TatRev
ruxolitinib 4μM	4	130 (2)	75	83	109	84 (2)
dinaciclib 8nM	6	202 (4)	83	76	92 (5)	0 (3)
AZD4573 8nM	6	249 (4)	73	71	87 (5)	0 (3)
pladienolide B 10nM	4	177 (2)	83	76	99	33
KRIBB11 2.5μM	3	144 (1)	73	86	97	368 (2)

C

D

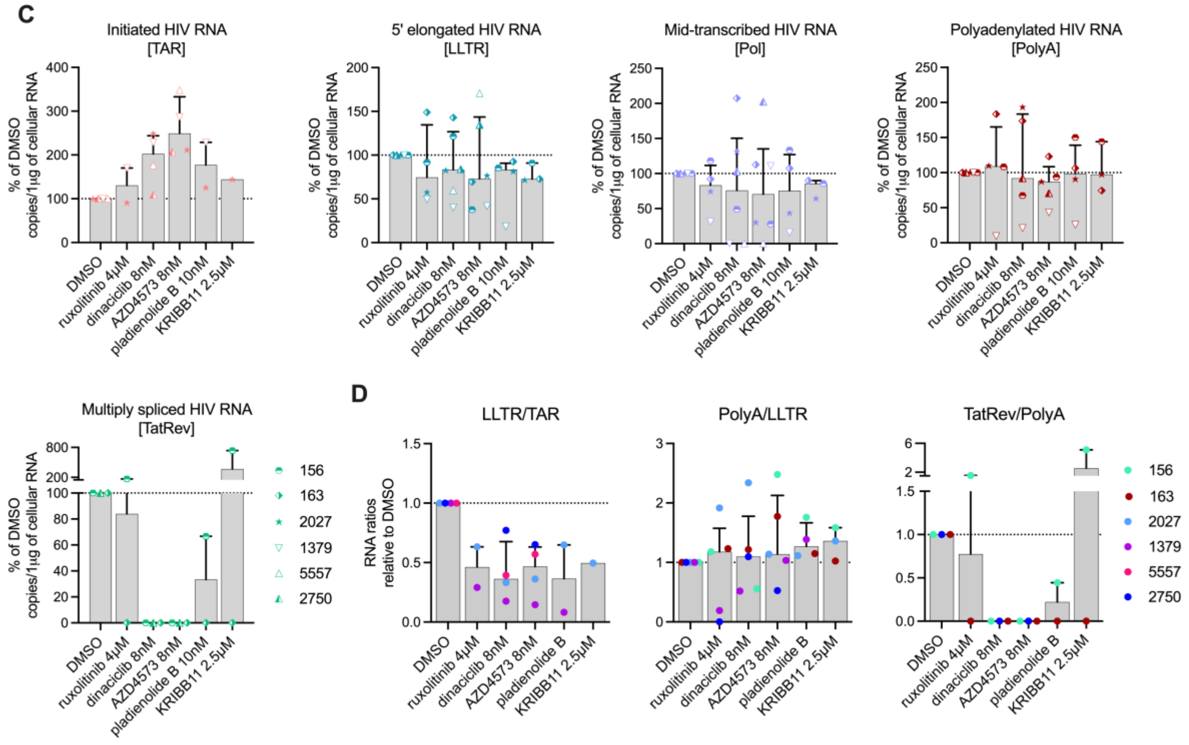

Figure S5. CDK-inhibitors appear to decrease baseline HIV splicing even in the absence of T cell activation. Each drug was tested in PBMCs from at least three ART-suppressed study participants and PBMCs were aliquoted into wells at 6x10^6 cells/well. The cells were cultured with antiretrovirals but without activation in the presence of individual drugs in

DMSO or DMSO alone as control. After 24h, total RNA was extracted and the progression through different stages of HIV transcription was quantified by measuring the levels of initiated (TAR), 5’ elongated (LLTR), mid-transcribed/unspliced (Pol), completed (PolyA) and multiply spliced (TatRev) HIV transcripts. **A-B)** The levels of all HIV transcripts were quantified, normalized to 1 µg of total cellular RNA, and expressed as a percent of the DMSO control (% of DMSO). **A)** Medians are shown, as well as the values of the individual study participants in different colors. **B)** The medians from panel A) are presented. The effect of each drug on the different transcripts was studied in three to six study participants, as indicated in the second column, unless specified otherwise in parentheses. **C)** The levels of initiated (TAR), 5’ elongated (LLTR), mid-transcribed/unspliced (Pol), completed (PolyA) and multiply spliced (TatRev) HIV transcripts relative to the DMSO control (% of DMSO). Medians with IQR are shown, with varying symbol shapes indicating individual study participants. **D)** The effect of each drug on HIV transcriptional progression was analyzed by the ratio of one HIV transcript to another. Ratios are independent of HIV infection frequency or normalization to cell numbers. Shown are the proportion of i) all HIV transcripts that were elongated [LLTR/TAR]; ii) elongated HIV transcripts that were completed [PolyA/LLTR]; and iii) completed transcripts that were multiply spliced [TatRev/PolyA]. Medians and IQR are presented, as well as the individual values per study participant in different colors.

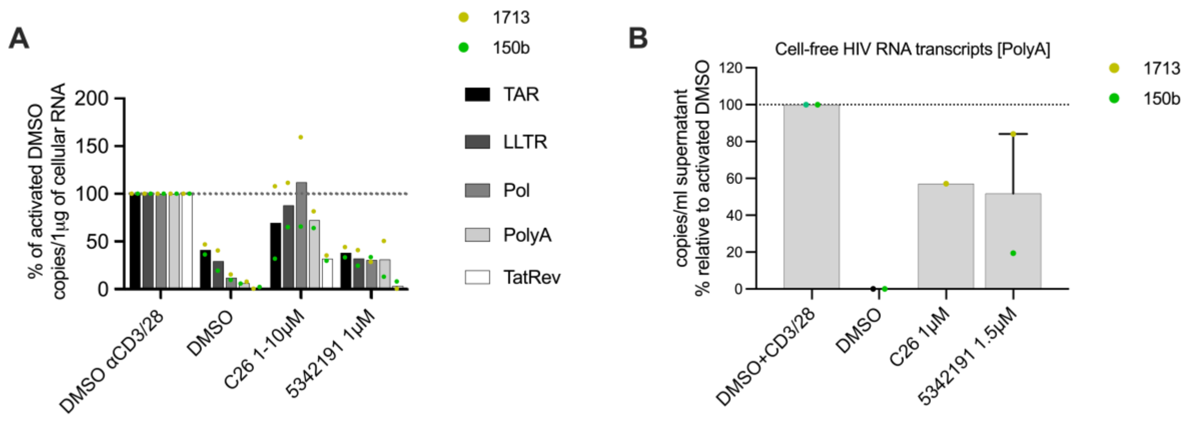

Figure S6. The effects of 5342191 and C26 on HIV transcriptional reactivation and viral RNA supernatant release at six days. PBMCs were isolated from ART-suppressed study participants and aliquoted into wells at $6x10^6$ cells/well. After activation, the cells were cultured with antiretrovirals in the presence of individual drugs in DMSO or DMSO alone as control. **A)** After six days, total RNA was extracted and the progression through different stages of HIV transcription was quantified by measuring the levels of initiated (TAR), 5' elongated (LLTR), mid-transcribed/unspliced (Pol), completed (PolyA) and multiply spliced (TatRev) HIV transcripts. The levels of all HIV transcripts were quantified, normalized to 1 µg of total cellular RNA, and expressed as a percent of the activated DMSO control (% of activated DMSO). Medians with IQR are shown, and different colours denote individual study participants. **B)** After six days, RNA was extracted from the culture supernatant and polyadenylated HIV RNA was quantified by RT-ddPCR as a measure of viral RNA in the supernatant and then normalized to the levels of the activated DMSO (% of activated DMSO). Medians and IQR are presented, as well as the individual values per study participant in different colors.

References

1. Gavegnano C, et al. Novel mechanisms to inhibit HIV reservoir seeding using Jak inhibitors. *PLoS Pathog* 2017;13(12):e1006740.
2. Gavegnano C, et al. Ruxolitinib and Tofacitinib Are Potent and Selective Inhibitors of HIV-1 Replication and Virus Reactivation In Vitro. *Antimicrob Agents Chemother* 2014;58(4):1977–1986.
3. Marconi VC, et al. Randomized Trial of Ruxolitinib in Antiretroviral-Treated Adults With Human Immunodeficiency Virus. *Clinical Infectious Diseases* 2022;74(1):95–104.
4. Ying H, et al. Selective Histonedeacetylase Inhibitor M344 Intervenes in HIV-1 Latency through Increasing Histone Acetylation and Activation of NF-kappaB. *PLoS One* 2012;7(11).
5. Lacombe B, et al. Specific Inhibition of HIV Infection by the Action of Spironolactone in T Cells. *J Virol* 2016;90(23):10972–10980.
6. Mori L, et al. The XPB Subunit of the TFIIH Complex Plays a Critical Role in HIV-1 Transcription, and XPB Inhibition by Spironolactone Prevents HIV-1 Reactivation from Latency. *J Virol* 2021;95(4).
7. Zhang HS, et al. Resveratrol inhibited Tat-induced HIV-1 LTR transactivation via NAD+-dependent SIRT1 activity. *Life Sci* 2009;85(13–14):484–489.
8. Harrich D, Jin H. RNA glycosidase and other agents target Tat to inhibit HIV-1 transcription. *Biochemical Journal* 2018;475(6):1059–1062.
9. Noel P, et al. Triptolide and Its Derivatives as Cancer Therapies. *Trends Pharmacol Sci* 2019;40(5):327–341.
10. Noel P, et al. Triptolide targets super-enhancer networks in pancreatic cancer cells and cancer-associated fibroblasts. *Oncogenesis* 2020;9(11).
11. Wan Z, Chen X. Triptolide inhibits human immunodeficiency virus type 1 replication by promoting proteasomal degradation of Tat protein. *Retrovirology* 2014;11(1):1–13.
12. Lv T, et al. Therapeutic effect of (5R)-5-hydroxytriptolide (LLDT-8) in SIV infected rhesus monkeys. *Int Immunopharmacol* 2022;110.
13. Wong RW, et al. Digoxin Suppresses HIV-1 Replication by Altering Viral RNA Processing. *PLoS Pathog* 2013;9(3).
14. Laird GM, et al. A novel cell-based high-throughput screen for inhibitors of HIV-1 gene expression and budding identifies the cardiac glycosides. *Journal of Antimicrobial Chemotherapy* 2014;69(4):988–994.

15. Wong RW, et al. Characterization of novel inhibitors of HIV-1 replication that function via alteration of viral RNA processing and rev function. *Nucleic Acids Res* 2013;41(20):9471–9483.
16. Toure MA, Koehler AN. Addressing Transcriptional Dysregulation in Cancer through CDK9 Inhibition. *Biochemistry* 2023;62(6):1114–1123.
17. Kawano T, et al. Activators and inhibitors of protein kinase c (Pkc): Their applications in clinical trials. *Pharmaceutics* 2021;13(11):1–27.
18. Jurkowska K, et al. Sirtuins as interesting players in the course of hiv infection and comorbidities. *Cells* 2021;10(10).
19. Giacca M. HIV Latency TORn Down. *Cell Host Microbe* 2016;20(6):700–702.
20. Pasquereau S, Herbein G. CounterAKTing HIV: Toward a “Block and Clear” Strategy?. *Front Cell Infect Microbiol* 2022;12.
21. Konrad T. Howitz, et al. Small molecule activators of sirtuins extend Saccharomyces cerevisiae lifespan. *Nature* 2003;425(6954):191–196.
22. Marzio G, et al. HIV-1 Tat transactivator recruits p300 and CREB-binding protein histone acetyltransferases to the viral promoter. *Proc Natl Acad Sci U S A* 1998;95(23):13519–13524.
23. Rose K, Bell L, Jacob S. Specific inhibition of chromatin-associated poly(A) synthesis in vitro by cordycepin 5’ -triphosphate . *Nature* 1977;267.
24. Kotake Y, et al. Splicing factor SF3b as a target of the antitumor natural product pladienolide. *Nat Chem Biol* 2007;3(9):570–575.
25. Eskens FALM, et al. Phase I pharmacokinetic and pharmacodynamic study of the first-in-class spliceosome inhibitor E7107 in patients with advanced solid tumors. *Clinical Cancer Research* 2013;19(22):6296–6304.
26. O’Brien K, et al. The biflavonoid isoginkgetin is a general inhibitor of pre-mRNA splicing. *Journal of Biological Chemistry* 2008;283(48):33147–33154.
27. Hasegawa M, et al. Identification of SAP155 as the target of GEX1A (Herboxidiene), an antitumor natural product. *ACS Chem Biol* 2011;6(3):229–233.
28. Andrew Timmons, et al. HSF1 inhibition attenuates HIV-1 latency reversal mediated by several candidate LRAs In Vitro and Ex Vivo. *PNAS* 2020;117(27):15763–15771.
29. Joshi P, Stoddart CA. Impaired infectivity of ritonavir-resistant HIV is rescued by heat shock protein 90. *J Biol Chem* 2011;286(28):24581–24592.
30. Anderson I, et al. Heat shock protein 90 controls HIV-1 reactivation from latency. *Proc Natl Acad Sci U S A* 2014;E1528–E1537.
31. Kim H, et al. Inhibition of HIV-1 reactivation by a telomerase-derived peptide in a HSP90-dependent manner. *Sci Rep* 2016;6:28896.

32. Sarah Pierson. The effect of a specific Heat Shock Protein 90 inhibitor on HIV-1 infection. Unpublished doctoral dissertation. George Mason University.2020;
33. Choi E, et al. Modulation of specificity protein 1 by mithramycin A as a novel therapeutic2014;1–8.
34. Hotter D, et al. IFI16 Targets the Transcription Factor Sp1 to Suppress HIV-1 Transcription and Latency Reactivation. *Cell Host Microbe* 2019;25(6):858-872.e13.
35. Wang J, et al. Retinoblastoma binding protein 4 represses HIV-1 long terminal repeat-mediated transcription by recruiting NR2F1 and histone deacetylase. *Acta Biochim Biophys Sin (Shanghai)* 2019;51(9):934–944.
36. Khalil BD, et al. An NR2F1-specific agonist suppresses metastasis by inducing cancer cell dormancy. *Journal of Experimental Medicine* 2021;219(1).
37. Weg-Remers S, et al. Regulation of alternative pre-mRNA splicing by the ERK MAP-kinase pathway. *EMBO Journal* 2001;20(15):4194–4203.
38. Wong RW, et al. An activator of G protein-coupled receptor and MEK1/2-ERK1/2 signaling inhibits HIV-1 replication by altering viral RNA processing. *PLoS Pathog* 2020;16(2).
39. Telwatte S, et al. Gut and blood differ in constitutive blocks to HIV transcription, suggesting tissue-specific differences in the mechanisms that govern HIV latency. *PLoS Pathog* 2018;14(11).
